# Protein-protein interaction as a predictor of subcellular location

**DOI:** 10.1186/1752-0509-3-28

**Published:** 2009-02-25

**Authors:** Chang Jin Shin, Simon Wong, Melissa J Davis, Mark A Ragan

**Affiliations:** 1 The University of Queensland, Institute for Molecular Bioscience, and ARC Centre of Excellence in Bioinformatics, QLD 4072, Australia

## Abstract

**Background:**

Many biological processes are mediated by dynamic interactions between and among proteins. In order to interact, two proteins must co-occur spatially and temporally. As protein-protein interactions (PPIs) and subcellular location (SCL) are discovered *via *separate empirical approaches, PPI and SCL annotations are independent and might complement each other in helping us to understand the role of individual proteins in cellular networks. We expect reliable PPI annotations to show that proteins interacting *in vivo *are co-located in the same cellular compartment. Our goal here is to evaluate the potential of using PPI annotation in determining SCL of proteins in human, mouse, fly and yeast, and to identify and quantify the factors that contribute to this complementarity.

**Results:**

Using publicly available data, we evaluate the hypothesis that interacting proteins must be co-located within the same subcellular compartment. Based on a large, manually curated PPI dataset, we demonstrate that a substantial proportion of interacting proteins are in fact co-located. We develop an approach to predict the SCL of a protein based on the SCL of its interaction partners, given sufficient confidence in the interaction itself. The frequency of false positive PPIs can be reduced by use of six lines of supporting evidence, three based on type of recorded evidence (empirical approach, multiplicity of databases, and multiplicity of literature citations) and three based on type of biological evidence (inferred biological process, domain-domain interactions, and orthology relationships), with biological evidence more-effective than recorded evidence. Our approach performs better than four existing prediction methods in identifying the SCL of membrane proteins, and as well as or better for soluble proteins.

**Conclusion:**

Understanding cellular systems requires knowledge of the SCL of interacting proteins. We show how PPI data can be used more effectively to yield reliable SCL predictions for both soluble and membrane proteins. Scope exists for further improvement in our understanding of cellular function through consideration of the biological context of molecular interactions.

## Background

Life at the cellular level of organization can be represented as an intricate and dynamic system of interactions among molecules. Proteins are fundamentally involved in all cellular processes, with many of their functions transduced through pairwise or multivalent interactions with other proteins. Discovering and modelling this network of protein-protein interactions (PPIs) has long been a goal of functional biology. Eukaryotic cells contain a number of physical compartments, across and within which their PPI networks are organised and structured. Each type of compartment provides a unique physiological environment within which specialized functions are carried out. To interact, proteins (or any other molecules) must necessarily share a common subcellular location (SCL), or an interface between physically adjacent SCLs, at least transiently or conditionally. Thus identifying the compartment(s) within which each protein is located,*i.e*. its SCL, is an important step in understanding its specific role in cellular physiology, and indeed in modelling this physiology more generally.

Both experimental and computational approaches have been applied to identify the SCL of proteins. Each has its own advantages and limitations. Experimental approaches are direct but typically time-consuming, expensive, potentially subject to artefact, and do not project the full range of biological complexity [1], whereas computational methods are potentially faster and more-general, may be less-accurate [2], and ultimately require empirical validation.

Recent studies based on high-throughput technologies have confirmed that interacting proteins tend to be located within the same compartment, or in physically adjacent compartments, in human [3-5], fly [6] and yeast [7,8]; Schwikowski *et al*. report that 76% of interactions in their yeast PPI set are between proteins located in the same SCL [7], while a review of human PPIs based on public databases and literature curation found 52% to involve co-located proteins plus others involving adjacent compartments [9]. These studies strengthen the assertion that a pair of interacting proteins is more likely to be co-located in the eukaryotic cell.

For decades, molecular biologists have discovered individual protein interactions using low-throughput techniques, accumulating low-coverage but high-quality PPI data. More recently these methods have been adapted, and new methods devised, to survey PPI on a more global, proteome-wide scale. However, these high-throughput methods have been reported to have high rates of false positives (FPs), and concerns exist that experimental conditions in these assays may not accurately reflect cellular physiology [6,10-13]. A high FP rate in PPI would of course undermine the utility of PPI data in predicting SCL, and consequently in modelling cellular physiology.

To improve the quality of PPI data, additional criteria have been examined including discovery by different analytical technologies, e.g. yeast two-hybrid (Y2H) assays and co-immunoprecipitation (co-IP) [13] or different affinity-purification protocols [14], co-expression of mRNAs [13,15], synthetic lethality [13,15], presence of homologs that interact in other species (interologs) [16] or of protein domains known to interact [16], and common functional annotation as reflected in keywords from Gene Ontology [17]. Statistical confidence can be assigned to interactions [15], and details of the local PPI network may be used to distinguish between true and false interactions [18]. von Mering and colleagues concluded that "as many complementary methods as possible should be used" to increase coverage and improve reliability [13]. Computationally derived features can be applied as well, e.g. the presence and nature of targeting and sorting signals, protein motifs, and transmembrane organization [19-22]. These criteria can be combined, weighted, and used in prediction tools, including in the prediction of SCL [22]. However, much remains to be done in extending these or similar approaches beyond yeast and human, e.g. to systems where more or different subcellular compartments may be recognised and PPI networks are less well-characterised; and to deal better with the approximately 20% of cellular proteins that are membrane-localised, hence less well-suited to study using most high-throughput experimental technologies. For this, it is necessary to identify, understand and quantify the individual factors that contribute to complementarity among different analytical and computational approaches.

Here we examine the contribution of six such factors to the inference of SCL from public PPI data in human, mouse, fly and yeast. We generate a high-confidence dataset for each species, and use it to test whether PPIs are in fact co-located: six publicly available PPI datasets were evaluated and integrated, while SCL information was obtained from Gene Ontology (GO) annotations in UniProt. To reduce the effect of false positive PPIs we generate six subsets of our data, each supported by a different line of evidence: three related to the evidence type itself (number of supporting detection methods, literature, and data sources), and three based on inferred biology (similarity of biological process, domain interactions, and interologs). We develop four alternative strategies and algorithms for using these PPI data to predict SCL, drawing explicitly on these six lines of evidence singly and in combination. Considering membrane and non-membrane proteins separately, we test these strategies against a set of newly released, manually curated SCL data not previously available to us, and compare the performance of our method to four previously published SCL prediction methods.

## Results and discussion

### 1. PPI data comparisons on proteins and interactions among databases

Inconsistency of annotation systems for newly discovered proteins at the different protein-sequence databases often results in different identifiers being assigned to the same protein [23]. To facilitate consistency in protein annotation, UniProt was launched in cooperation with three major protein sequence databases: Swiss-Prot, TrEMBL and PIR [24]. For consistent integration of different PPI databases, we mapped identifiers (IDs) used by each database to the corresponding UniProt accession number (AC), giving us a standard identifier over our integrated dataset.

**Figure 1:**
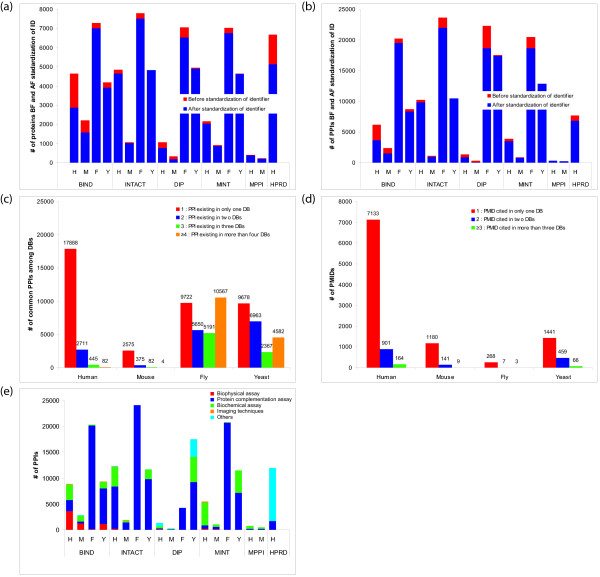
**Comparison of PPI databases**. (a) Numbers of proteins in each PPI database. The bars indicate, for each of the four species, the total number of unique proteins in the six databases (DBs) before (BF: black) and after (AF: grey) standardization of identifiers (IDs). (b) Numbers of PPIs in each database before (black) and after (grey) ID standardization. (c) Numbers of PPIs present in one, two, three, or four or more databases after redundancy reduction. Human PPIs have been accessioned into all six databases, mouse PPIs into five, and fly and yeast PPIs into four. The counts include homo- as well as hetero-dimeric interactions. (d) Numbers of PPIs for which one, two, or three or more distinct literature items are cited as evidence. (e) Numbers of PPIs supported by the four high-level experimental approaches. For a-e: H (human), M (mouse), F (fly) and Y (yeast).

After standardization, a number of identifiers in each database remained unmapped to UniProt AC; we omitted these proteins from our integrated dataset. Redundant, divergent and convergent entries were also removed. With the human and mouse data, this process resulted in a substantial loss of proteins from some sources (for example, in BIND, DIP and HPRD, between 23–47% of human and mouse proteins were removed), while other resources showed much smaller reductions in protein number (4–6%). In the fly and yeast data, fewer proteins were redundant, or failed to map to UniProt ACs; only 4–8% of fly proteins and 0.2–7% of yeast proteins were removed across databases. Many of these difficulties in mapping Genbank Identifiers, NCBI Reference Sequence (RefSeq) ACs, and Protein Sequence Database (PSD) identifiers unambiguously to UniProt ACs were due to changes in annotation convention at the public databases, and/or from intrinsic complexity of the data, and highlight a continuing issue with integration among protein sequence databases (Fig. 1a).

Removal of unmapped proteins and resolution of redundant proteins resulted in the removal of 12% of PPIs from BIND, 5% from IntAct, 11% from DIP, 6% from MINT, 12% from MPPI and 12% from HPRD. As expected from the results of protein mapping described above, PPI numbers for human and mouse were substantially reduced from the BIND, DIP and HPRD data. For example, in BIND, 42% of human PPIs and 37% of mouse PPIs were removed, while in DIP, 34% of human PPIs and 65% of mouse PPIs were removed (Fig. 1b).

### 2. Biases among PPI databases

We compared several properties of PPI datasets across databases to search for substantial biases in these data. Parameters considered include the number and overlap of proteins and PPIs recorded in databases, literature citations attributed to PPIs, and experimental methods for PPI detection. We consider these factors important to establish the independence of the data contained in each resource, hence the quality of our confidence measures. For example, our measure of the multiplicity of records in databases would be undermined if the multiple entries actually represent a single observation from one paper that had been shared among databases that frequently exchange data , whereas if they had been identified from different papers they would represent independent observations. For this and other measures of confidence, analysis of overlapping PPIs and literature provides an important measure of reliability. To inspect this overlap, we interrogated the intersections of PPIs and PMIDs collected among databases, and compared the types of experimental methods used to discover PPIs.

#### 2.1 Intersections of PPIs among PPI databases

Despite similar objectives and approaches among PPI databases [25], each resource follows its own process for identifying relevant literature and extracting interaction data. To analyse the heterogeneity of PPI collections, we examined PPIs that are common among databases. For all the species considered, most PPIs are recorded in only one database (Fig. 1c). About 85% of human and mouse, 31% of fly and 41% of yeast PPIs in our dataset exist in only one database. No human PPI was common to all six databases that contain human data, and no mouse PPI was common to all five databases that contain mouse data. However, around 30% of fly and 20% of yeast PPIs were common to all four databases that contain PPIs from those species (BIND, IntAct, DIP and MINT). The greater overlap in fly and yeast PPIs is a consequence of the large-scale interaction studies published for these species [6,26,27]. For fly, the large-scale interaction map of Giot *et al*. [6] identified around 20,000 protein interactions, including 4700 high-confidence interactions, which appeared in all four relevant PPI databases, while Formstecher *et al*. [28] detected 2300 interactions that are referenced in IntAct, BIND and DIP. Three large-scale PPI maps of yeast have also been incorporated into the four relevant databases [29-31].

#### 2.2 Intersections of literature cited for PPIs

To examine the literature shared in common by these databases, we used PubMed identifiers (PMIDs), which are unique identifiers for biomedical literature, as a proxy for journal articles. We found little overlap of PMIDs among these six PPI databases. Articles which are cited in only one database covered 87% of human, 89% of mouse, 96% of fly and 73% of yeast PPIs (Fig. 1d). Across all these species, only 1–3% of papers are referenced by more than three different databases; thus the curation and retrieval systems of these databases differ significantly with respect to this outcome. Interestingly, although at the time of analysis the fewest papers were cited for fly, its interaction data were more numerous than for any other species (Fig. 1d), reflecting the dominance of large-scale proteome-wide interaction studies for this species.

#### 2.3 Supporting methods for PPIs among databases

We categorized PPI-detection methods into classes based on the terminology provided by the Protein Standards Initiative (PSI) Molecular Interaction (MI) vocabulary [32] (Fig. 1e). The four main classes are Biophysical assay (MI:0013), Biochemical assay (MI:0401), Protein complementation assay (MI:0090), and Imaging techniques (MI:0428); we collected other methods (*e.g*. Experimental detection, MI:0045) into an Others class. The supporting method most frequently identified in these data was protein complementation, within which yeast two-hybrid (Y2H) methods [33] were the most frequently cited: more than 90% of fly PPIs were identified by Y2H methods (MI:0018 and MI:0398). The next most-frequent methods were biochemical, including affinity techniques [34].

Most PPIs in these databases have been identified using complementation assays, reflecting the continuing dominance of Y2H techniques for high-throughput PPIs determination. Individual exceptions exist, *e.g*. a large proportion of human and mouse PPIs in the BIND database identified by biophysical methods, where x-ray crystallography (MI:0114) alone represents around 30% of all PPIs; this reflects the inclusion in BIND of data from structural studies of protein complexes. Given the doubts that currently exist about the rate of FP predictions in high-throughput data [35], the substantial contribution of Y2H studies to these public databases requires us to filter the data in generating a high-confidence PPI dataset, and thereafter to remain cautious in using such sets for inferring SCL.

#### 2.4 Summary of bias analysis

Given the low degree of overlap among PPI sets from different databases especially for human and mouse, it appears worthwhile to integrate data from multiple resources to maximize coverage of the interactome. Although for fly and yeast more PPIs are common to more databases (Fig. 1c), the actual overlap in the underlying literature is much lower (Fig. 1d). For human and mouse, overlap of protein sets among these databases does not equate to a high proportion of overlapping PPIs: most interactions are unique to a single database. The situation is different for the yeast and fly data, where significant high-throughput or genome-wide PPI analysis has resulted in a greater proportion of interacting pairs present in several databases (Fig. 1c,d).

With the latter species especially, approaches that base confidence in a PPI on its co-occurrence in separate databases are misdirected, as presence in multiple databases reflects the accession of a few papers reporting large-scale studies, rather than truly independent observations of the interaction captured from unrelated literature.

### 3. Comparison of data on subcellular location

#### 3.1 Coverage of GO Cellular Component annotations in the PPI dataset

To investigate subcellular location, we extracted from UniProt the GO Cellular Component (CC) annotations for proteins in our dataset. To optimize reliability of the annotation, GO terms with evidence codes IC (Inferred by Curator), IEA (Inferred from Electronic Annotation), ISS (Inferred from Sequence or Structural Similarity), NAS (Non-traceable Author Statement), ND (No biological Data available), and NR (Not Recorded) were not included due to the uncertain provenance of these annotations (see Methods, Section 1.3). GO is an ongoing project, and the assignment of terms to proteins can be subjective: for example, some proteins are annotated with a more-general term higher in the GO hierarchy, whereas others are annotated with lower-level, more-specific terms. For consistency, we collapsed all CCs for each protein to the parent terms in our definition of cellular compartments. In this way we associated proteins to one or more of 15 compartments (Methods, Section 2.1).

**Figure 2:**
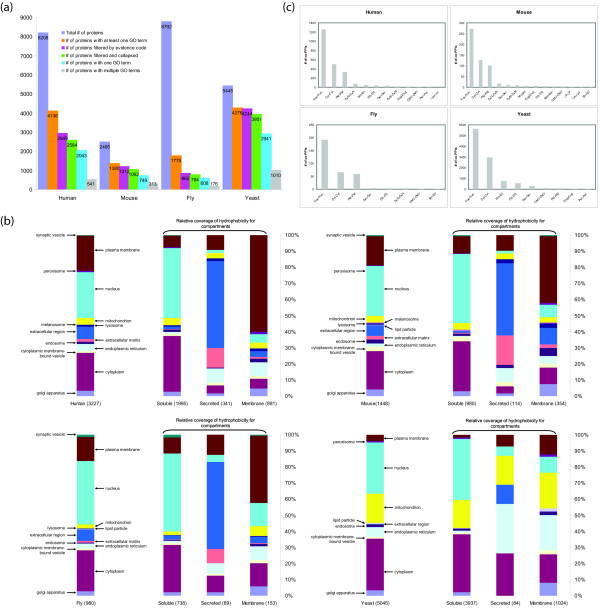
**Subcellular location of proteins and PPIs**. (a) Numbers of proteins with at least one Gene Ontology (GO) term for cellular component (CC). For each species, the six bars from left to right represent the numbers of proteins (1) in our in-house dataset (purple), (2) with at least one GO CC (orange), (3) with a GO CC term after filtration based on evidence code (4) with a GO term after collapse of terms (green), (5) with only one GO term (blue), and (6) with multiple GO terms (grey). Bars (5) and (6) are numbers after filtration based on evidence and collapse of GO terms. (b) Proportional distribution of protein numbers according to cellular compartment. Proteins are counted once in each annotated location; those with multiple locations have been counted a corresponding number of times. The bar on the far left represents overall proportion of compartment. The other three bars represent soluble (left), soluble secreted (middle), and membrane (right) proteins. GO CC terms were recorded after quality filtration and collapse. (c) For each species, numbers of co-PPIs in the three most-abundant CC locations. Both interacting proteins are required to have least one GO CC term. For a-c: CMV (cytoplasmic membrane-bound vesicle), Cyt (cytoplasm), End (endosome), ER (endoplasmic reticulum), ExM (extra cellular matrix), ExR (extra cellular region), Gol (golgi apparatus), LP (lipid particle), Lys (lysosome), Mel (melanosome), Mit (mitochondrion), Nuc (nucleus), Per (peroxisome), PM (plasma membrane), SV (synaptic vesicle).

After sorting GO terms according to evidence codes and collapsing terms, 32% of human proteins, 43% of mouse proteins, 9% of fly proteins and 73% of yeast proteins in our combined dataset are associated with one or more GO CC terms. For these four species respectively there are 17%, 27%, 2% and 71% heterodimeric interactions in which each partner has at least one GO CC term (Fig. 2a). In about half of these interactions (9%, 12%, 1% and 40% respectively), each interacting protein is associated with exactly one GO CC term, i.e. is annotated as being located in only one of our high-level cellular compartments.

To assess the potential for PPI-based inference to uncover new subcellular location information, we identified heterodimeric PPIs in which only one interacting partner (but not both) is annotated with at least one GO CC term. In these four species we found 8830, 1239, 5775 and 5727 heterodimeric PPIs that satisfy this criterion. These are our potential prediction sets, and contain 3280, 783, 2998 and 1406 unique proteins that lack SCL annotation. The missing SCL annotation could potentially be inferred from the known location of the interaction partner(s) (Additional file 1).

#### 3.2 Cellular compartments enriched for proteins with GO CC annotations

In our combined data set, we examined the distribution of proteins according to their annotated subcellular locations. The most frequent annotations are for location in the nucleus, cytoplasm, plasma membrane and extracellular region (Fig. 2b). Across all species, 40–50% of proteins with multiple SCL annotations have locations annotated in both nucleus and cytoplasm. Additionally, around 10–20% of multiply located proteins in the three morphologically complex eukaryotes are annotated with locations in the cytoplasm and plasma membrane, while for yeast 13% of multiply located proteins are located in the cytoplasm and mitochondrion.

We applied a previously published method [36] based on hydrophobicity to predict soluble, secreted and integral membrane proteins, then examined the distribution of proteins in these three classes across compartments. About 93% and 88% of proteins from the PPI datasets with annotated locations in the nucleus and cytoplasm respectively were predicted to be soluble. The background proportion of soluble intracellular proteins across whole proteomes is usually around two-thirds [37], suggesting that soluble proteins participating in PPIs are enriched in the nucleus and cytoplasm in these organisms. This apparent enrichment may reflect biological reality, and/or be a consequence of the bias of protein complementation assays (in particular, Y2H) which favor soluble proteins. As described in Section 2.3, Y2H is a major source of PPIs in these datasets.

As expected, over half of the proteins annotated as located in the plasma membrane were predicted to be alpha-helical and integral to the membrane. Proteins annotated as located in the extracellular region were largely predicted to be either secreted or membrane proteins. In some cases, an integral membrane protein may be annotated as extracellular because a significant part of it has been detected there experimentally. Proteins in the endoplasmic reticulum (ER) were predicted as secreted or membrane proteins (Fig. 2b), likely reflecting the role of this organelle in the co-translational translocation of proteins in the secretory pathway: classically secreted proteins and alpha-helical membrane proteins are synthesized in the ER [38].

### 4. Enrichment of co-located PPIs in cellular compartments

We were interested in locations enriched for pairs of interacting proteins where each protein within a pair is annotated with that location (co-located PPIs, subsequently referred to as co-PPIs). For PPIs with proteins co-located in two or more compartments, we scored a co-PPI for each shared compartment. Most (48–58% across species) co-PPIs were located in the nucleus (Fig. 2c). The second most common place where interacting pairs of proteins were found to be located was the cytoplasm in all four species. The third most common location observed was the plasma membrane in human, mouse and fly, and the mitochondrion in yeast. One reason why interacting proteins are abundant in the nucleus is that large numbers of proteins so far have been examined in this organelle [39].

### 5. Co-location of PPIs in reference and random sets

We examined the degree to which interacting proteins are likely to share the same compartment, and compared this incidence of co-location to that computed for the same PPI set with randomized GO CC annotations. We first filtered GO CC terms based on evidence codes, then collapsed to higher-level GO CC terms representing fifteen subcellular locations (see Methods, Section 2.1).

Our combined dataset contains 1860 proteins and 3298 PPIs in human, 780 proteins and 740 PPIs in mouse, 461 proteins and 540 PPIs in fly, and 3837 proteins and 16110 PPIs in yeast. This reference dataset for each species is composed of PPIs that are non-redundant, heterodimeric interactions in which both interacting partners have at least one annotated GO CC term. The proportion of co-PPIs was calculated in the reference set in each species. Across the four species, in 57–69% of PPIs both interacting proteins share at least one GO CC term in common (Table 1).

**Table 1:** Co-located PPIs in reference and evidence-supported subsets

**PPI sets**	**Human**			**Mouse**			**Fly**			**Yeast**		
	
	**Total PPI (#)**	**Co-PPI (#)**	**Co-PPI (%)**	**Total PPI (#)**	**Co-PPI (#)**	**Co-PPI (%)**	**Total PPI (#)**	**Co-PPI (#)**	**Co-PPI (%)**	**Total PPI (#)**	**Co-PPI (#)**	**Co-PPI (%)**
**Reference sets**												
Known locations	3298	2115	64%	740	512	69%	540	310	57%	16110	9693	60%
Randomized locations	3298	1045	32%	740	268	36%	540	220	41%	16110	6118	38%
												
**Subsets**												
BPscore	366 (11%)	308	84%	79 (11%)	68	86%	65 (12%)	63	97%	3387 (21%)	3177	94%
DDI	512 (16%)	368	72%	158 (21%)	130	82%	49 (9%)	46	94%	806 (5%)	671	83%
Interologs	134 (4%)	114	85%	73 (10%)	56	77%	22 (4%)	20	91%	256 (2%)	234	91%
DB	581 (18%)	426	73%	105 (14%)	82	78%	264 (49%)	123	47%	9579 (59%)	5943	62%
Method	378 (15%)	280	74%	110 (16%)	75	68%	27 (5%)	24	89%	2879 (20%)	2329	81%
PMID	281 (9%)	213	76%	56 (8%)	46	82%	23 (4%)	21	91%	1706 (11%)	1462	86%
												
**Union of subsets**												
Biological evidence type (BIO)	838 (25%)	642	77%	253 (34%)	207	82%	101 (19%)	94	93%	3825 (24%)	3508	92%
Recorded evidence type (EVI)	793 (23%)	583	74%	216 (30%)	161	75%	295 (54%)	149	51%	9733 (60%)	6087	63%

* The proportion of the reference set covered by total PPIs in each subset is indicated in parentheses.

To test whether these observed frequencies of co-PPIs could occur by chance (given the data), we randomly shuffled GO CC terms for proteins in the reference set while keeping interacting pairs intact, and calculated co-PPI frequencies in these random sets. The latter proportions (32%, 36%, 41% and 38% for human, mouse, fly and yeast respectively: Table 1) are substantially smaller than in the original data. This strongly suggests that the observed frequencies of co-location would not arise by chance.

### 6. Co-location of interacting proteins high-quality datasets

To determine the likely efficacy of using PPI networks to improve the inference of SCL (*e.g*. by reducing FP rate), we investigated strategies for extracting subsets of the data that have higher proportions of co-PPIs. To this end we established a number of high-quality PPI datasets, and compared co-PPI frequencies in these sets to those observed in the reference and random sets described immediately above (Section 5). This allowed us to test the proposition that many PPIs in which the protein interactors are annotated as occurring in different SCLs are in fact false positive interactions.

#### 6.1 Evidence used to extract supported subsets of PPI data

The occurrence of false positives in PPI data has been widely reported [10-13], and FP interactions are doubtlessly present in our reference datasets. To generate a PPI dataset of the highest possible quality, we assessed six lines of additional evidence that might add confidence that PPIs are indeed true positives. We examined how interacting pairs of proteins are supported by the following six lines of evidence:

i. similarity of GO Biological Process (BP) terms, as represented by the BP score [40];

ii. presence of domains known to participate in domain-domain interactions (DDIs) [41];

iii. knowledge of equivalent interactions between orthologs (interologs) in alternative species [42,43];

iv. accession into multiple databases;

v. support by more than one literature reference; and

vi. discovery using multiple experimental detection methods.

The final three types of supporting evidence (iv-vi) are different approaches to estimating whether multiple independent observations underlie a given PPI, but are not necessarily independent of each other. Entries in multiple databases might reference the same paper; interactions supported by multiple citations might represent a single original observation referred to in an additional context, or replicate a result using the same experimental method and therefore potentially suffer the same likelihood of FP prediction as the original discovery; and multiple experimental methods might be used within a single laboratory or paper but nonetheless be based on the same construct re-used in different methodological contexts. Keeping these caveats in mind, we examined the usefulness of these evidence types as proxies for multiple independent observations.

##### (i) Interactions supported by BP score

A biological process can be considered to be a series of functions or events. Many of these events involve PPIs, and it can be imagined that two interacting proteins are likely to participate in the same biological process (BP). Ewing and colleagues [3] found that shared GO BP annotations are significantly enriched in sets of interacting proteins. To assess the similarity of GO BP terms between interacting partners in our data, we used FSST, a local version of the web-based GOTaxExplorer [40] to calculate the semantic similarity of BP terms for pairs of interacting proteins. FSST compares the occurrence of GO terms against expectation based on their frequency in the database [40]. Lowest common ancestor (LCA) terms are selected for all combinations of GO terms assigned to interacting proteins, and a score is computed based on a combination of the Resnik [44] and Lin [45] measures. We consider PPIs with a BP score over 0.70 to be *supported by evidence of shared biological process*.

##### (ii) Interactions supported by DDI

A protein interaction requires physical contact and/or chemical reaction between subsets of amino acid residues in two or more protein molecules. These contacts or reactions are thought to be largely mediated by structural domains: domain-domain interactions (DDIs) underpin PPIs. We used iPfam, a subset of the Pfam database of protein domains and families, as our source of interactions between Pfam domains that have a representative structure deposited in Protein Data Bank (PDB) [46]. We obtained Pfam domain predictions from UniProt for our protein sequences, then consulted iPfam to identify sets of potentially interacting domains in the domain architectures of the interaction partners. If at least one known DDI pair was present, that PPI was considered to be *supported by DDI evidence*.

##### (iii) Interactions supported by interologs

It has been proposed that pairs of interacting proteins are often conserved across species [42]. For a given interacting pair in one species (*e.g*. human), if orthologs are present in another species (*e.g*. mouse) and form an interaction pair there, then that second interaction pair (in this example, the pair in mouse) is referred to as the *interolog *of the interaction pair in human [47]. Lehner and Fraser [48] constructed a human protein interaction map based on conserved interactions among worm, fly and yeast. We obtained ortholog information from the Inparanoid database [49] and identified interologs among human, mouse, fly and yeast. An interaction was considered to be *supported by interolog evidence *if an interolog was identified in at least one other of these species.

##### (iv) Interactions supported by presence in multiple databases

Each PPI database has its own literature retrieval system, its own criteria for identifying and accepting PPIs, and different human experts who extract and curate PPI information from the literature. It is thus reasonable to suppose that PPIs accepted into multiple databases are those supported by broader or less-equivocal evidence. We consider that PPIs accessioned into more than one PPI database to be *supported by presence in multiple databases*.

##### (v) Interactions supported by literature

Several assessment systems for true positive interactions employ literature as supporting evidence. Rual *et al*. [4] extracted a core interaction set of PPIs mentioned in more than one item of literature (*e.g*. journal article). PubMed assigns each item a unique PubMed identifier (PMID). We asked whether, over all databases, a PPI is associated with only one, or more than one, PMID and in the latter case consider an interaction to be *supported by literature*. No special consideration was given to possible overlap of authors or institutions among multiple literature sources.

##### (vi) Interactions supported by experimental detection methods

PPIs observed under different experimental methods show low FP frequencies. Several interaction networks have been described based on the assumption that PPIs supported by different methods may be assigned as true positives [12,13,50]. For example, von Mering and colleagues [13] found that interactions supported by more than one method are more likely to be true positives than those supported by a single method, while Sprinzak *et al*. [12] report that the more detection methods used to identify PPIs, the higher the frequency of true positive interactions. We collapsed the PSI-MI descriptions of method to the high-level categories presented earlier, and consider PPIs annotated as identified by more than one high-level method to be *supported by experimental detection methods*.

### 6.2 Coverage of reference PPIs by subsets supported by additional lines of evidence

Using each of these lines of evidence in turn, we created six subsets from each of the four species-specific reference sets. Each of these 24 subsets was constituted from only those PPIs supported by one of the lines of evidence described above. We then observed the proportion of co-PPIs (number of co-PPIs/number of PPIs) in each subset. The number of proteins, and observed and expected proportions of co-PPIs, in each subset are presented in Table 1.

For 22 of the subsets, the proportion of co-PPIs vis-à-vis the parent reference set was increased; the exceptions were multiple database evidence in fly, and for multiple experimental methods in mouse. A higher proportion of co-PPIs in a subset than in the parent reference set implies that use of that evidence type can improve the inference of SCL. Note that this proportion places an upper bound on the degree of improvement that can be expected in similarly supported PPI sets where the location of one protein is unknown. An obvious initial question is which types of evidence are most efficacious in improving the inference of SCL.

#### (i) Interactions supported by BP score

We found that PPIs in which the interacting proteins are annotated participants in similar biological processes have higher rates of co-PPI than in the corresponding reference sets (Table 1). These subsets exhibit the highest proportion of co-PPIs of all subsets, except in the case of human PPI data, where the interolog-supported subset has a slightly higher proportion. The proportion of co-PPIs was increased by up to 40 percentage points in comparison with the reference sets, with the greatest increases in fly and yeast. We varied the threshold for BP score, and observed that the more-stringent the threshold, the greater the proportion of co-PPIs, although at the expense of reduced coverage of the parent set (see Additional file 2).

#### (ii) Interactions supported by DDI

Requiring each member of an interacting pair of proteins to contain a known interaction domain increased the proportion of co-PPIs by 8 to 37 percentage points across species, compared with the corresponding reference sets.

#### (iii) Interactions supported by interologs

Requiring each PPI to have an interolog in at least one of the other three species in this analysis increased the proportion of co-PPIs by 8 to 34 percentage points compared to the corresponding reference set. We did not require supporting interologs to be present in our reference set. Fewer than 10% of reference PPIs were supported by interologs, rendering this line of evidence the least generally applicable.

Where annotation is available, subcellular location of interologs is highly conserved across species. We examined co-PPIs in human for which at least one interolog has been identified in one of the other three species in this study, and SCL of both members of that interolog has been annotated: 85 such interolog pairs were found. Of these interologs, 79 (93%) are co-PPIs in the same SCL as their human counterpart, 4 share at least one SCL annotation term with their human counterpart but are not co-PPIs in the second species (3 mouse, 1 fly), and 1 is a co-PPI in mouse but in a different SCL than in human (see Additional file 3). Thus across phyletically diverse eukaryotes, co-PPIs (and, probably, PPIs more generally) tend to be found in the same SCL as their interologs. These results generalize the observation that for yeast nucleolar proteins with at least one homolog in human, about 90% of the human homologs are also nucleolar [51].

#### (iv) Interactions supported by presence in multiple databases

Most PPI subsets accepted for inclusion in multiple databases show between 62–78% of co-PPIs. The exception is for fly, where the predominance of a few large-scale Y2H studies accepted into multiple PPI databases resulted in a lower proportion of co-PPIs when compared with the fly reference set. In yeast, another organism with many high-throughput studies, only a very small increase in co-PPIs (62% from 60%) was observed. This indicates that the presence of a PPI interaction in multiple databases is a reliable indicator of quality only where the curated PPI data are not dominated by large-scale studies. In time, as more high-throughput studies are released for more organisms, this line of supporting evidence may become increasingly less appropriate as a means of establishing data quality.

We also considered PPIs which were generated at least one time in a small-scale manner. We define small-scale studies to be those for which the corresponding literature item (*e.g*. article) reports fewer than 10 PPIs (counts based on our in-house database). For this subset the proportion of co-PPIs is dramatically increased in fly and yeast, although the coverage of PPIs is greatly reduced. For human and mouse, however, restricting the analysis to small-scale studies did not significantly change the co-PPI proportion (see Additional file 4).

#### (v) Interactions supported by literature

When PPIs are required to have support from two or more unique PMIDs, the proportion of co-PPIs increases by 12–34 percentage points compared with values for the corresponding reference set. These increases compare favorably to those in the subsets supported by presence of interologs (above) except for human, where the increase is only 60% as great. Coverage, however was higher than observed for interolog-supported subsets.

#### (vi) Interactions supported by detection methods

PPIs identified using at least two high-level experimental detection methods showed a high proportion of co-PPIs (68–89%) in comparison with the corresponding reference sets. Biochemical assay methods (MI:0401) remain more widely applied than those based on protein complementation (MI:0090) such as yeast-two-hybrid (Y2H), although Y2H has been used in many studies and large-scale analyses (see Additional file 5).

### 7. Co-location of PPIs in unions of supported subsets

The above results demonstrate that use of additional lines of evidence almost always yields higher-quality interaction data as assessed by proportion of co-PPIs, and suggest strongly that some or all of these approaches might be mobilized in predicting the SCL of interacting proteins whose location is not already known or annotated. With few exceptions, however, requiring additional evidence greatly decreases the coverage, assessed as a fraction of known PPIs (Table 1). In an attempt to improve coverage (while preserving quality), we investigated the relationship between coverage and co-PPI proportion in unions of subsets (Table 1). We tested the union of biological evidence type (BIO: BPscore∪DDI∪interologs), and the union of recorded evidence type (EVI: DB∪PMID∪methods). The subsets supported by biological evidence type always showed a higher proportion of co-PPI than is seen for the subsets supported by recorded evidence type (3–42 percentage points higher, Table 1), indicating that these lines of evidence provide greater enrichment of co-located PPIs in our data sets. We also tested combinations involving both biological and recorded evidence types (results not shown).

For each of the four species, the union of biological evidence type yielded co-PPI proportions intermediate among those of the constituent approaches individually, but with a 3 (yeast) to 23 (mouse) percentage point improvement in coverage vis-à-vis the best single consistent approach. The union of recorded evidence type approaches likewise gave co-PPI proportions intermediate among those of the corresponding constituent approaches, and a 14 (human) to 50 (fly) percentage point improvement in coverage. Quality or coverage (not both simultaneously) could be further increased by optimising the combination of biological and recorded evidence type.

### 8. Formalization and evaluation of prediction method

To evaluate more formally the potential benefit of using PPI data to predict SCL, we formalized several variants of our approach, then evaluated these variants on the species-specific reference sets, the six subsets described above, and selected unions of subsets (Table 2). Recall that all protein interactors are consistently annotated with a GO CC term based on UniProt, and that redundant PPIs have been removed. Our four variants, DISCRETE, MERGED, COMMON and MAJORITY, differ in the GO CC termset associated with each protein.

**Table 2:** Evaluation of prediction method variants on human reference and supported subsets

**PPI sets**	**DISCRETE**				**MERGED**				**COMMON**				**MAJORITY**			
	
	**PA**	**SA**	**PA** **(M)**	**SA** **(M)**	**PA**	**SA**	**PA** **(M)**	**SA** **(M)**	**PA**	**SA**	**PA** **(M)**	**SA** **(M)**	**PA**	**SA**	**PA** **(M)**	**SA** **(M)**
Reference	0.64	0.35	0.50	0.27	0.72	0.26	0.62	0.23	0.65	0.40	0.53	0.32	0.68	0.34	0.58	0.27
BPscore	0.84	0.56	0.76	0.46	0.86	0.55	0.79	0.42	0.85	0.57	0.77	0.47	0.86	0.55	0.79	0.43
Interologs	0.85	0.58	0.70	0.46	0.84	0.54	0.69	0.42	0.83	0.57	0.67	0.45	0.84	0.56	0.69	0.45
DDI	0.72	0.46	0.65	0.43	0.75	0.43	0.70	0.42	0.72	0.48	0.66	0.49	0.74	0.45	0.69	0.44
PMID	0.76	0.46	0.60	0.28	0.76	0.42	0.60	0.27	0.74	0.45	0.56	0.30	0.76	0.43	0.59	0.28
Method	0.74	0.38	0.61	0.29	0.76	0.37	0.63	0.27	0.73	0.41	0.58	0.32	0.75	0.38	0.62	0.29
DB	0.73	0.37	0.53	0.25	0.74	0.35	0.61	0.26	0.71	0.40	0.57	0.30	0.73	0.37	0.59	0.28
ALL	0.74	0.42	0.61	0.35	0.78	0.38	0.69	0.33	0.74	0.45	0.64	0.40	0.76	0.41	0.67	0.36
BIO	0.77	0.49	0.67	0.43	0.79	0.45	0.71	0.38	0.77	0.50	0.67	0.45	0.78	0.47	0.70	0.41
EVI	0.74	0.37	0.55	0.25	0.75	0.34	0.62	0.25	0.71	0.41	0.57	0.30	0.74	0.37	0.61	0.27

ALL: Union of all subsets (BPscore ∪ Interologs ∪ DDI ∪ PMID ∪ Method ∪ DB)

BIO: Union of biological evidence type subsets (BPscore ∪ Interologs ∪ DDI)

EVI: Union of recorded evidence type subsets (PMID ∪ Method ∪ DB)

M: Interactions involving an integral membrane protein

The permissive accuracy (PA) and strict accuracy (SA) were calculated for all variants (DISCRETE, MERGED, COMMON and MAJORITY) for all interactions, and for interactions involving an integral membrane protein (M).

Consider dataset **D** consisting of five proteins (A-E) and five PPIs, with an interaction denoted by the colon (:) and "true" SCLs (in parentheses) based on UniProt:

A (nuc) : B (nuc)

A (nuc) : D (nuc+cyto)

B (nuc) : C (nuc)

B (nuc) : D (nuc+cyto)

D (nuc+cyto) : E (nuc)

Note that X : Y is equivalent to Y : X. First, we select at random one protein from each PPI in **D** and temporarily mask its annotated location. We denote these proteins using the prime ('), and the resulting dataset as** D**':

A' (--) : B (nuc)

A' (--) : D (nuc+cyto)

B' (--) : C (nuc)

D' (--) : B (nuc)

E' (--) : D (nuc+cyto)

We then proceed as follows for the four variants (DISCRETE, MERGED, COMMON and MAJORITY). In the DISCRETE variant, we simply replace the masked GO CC term with the known location of the interaction partner:

A' = nuc from B (nuc)

A' = nuc+cyto from D (nuc+cyto)

B' = nuc from C (nuc)

D' = nuc from B (nuc)

E' = nuc+cyto from D (nuc+cyto)

To evaluate the performance of the method, we then compare these newly assigned term(s) with the true GO CC term(s) from dataset **D**.

By contrast, in the MERGED variant (given dataset **D** and the randomized dataset **D**' as above), for each masked protein (not PPI) in turn, the GO CC term(s) are assigned based on the union of SCLs annotated for all its interaction partner(s) in dataset **D**':

A' : B and A' : D; from B (nuc) and D (nuc+cyto), A' = nuc+nuc+cyto = nuc+cyto

B' : C; from C (nuc), B' = nuc

D' : B and D' : E; from B (nuc) and E (nuc), D'= nuc+nuc = nuc

E' : D; from D (nuc+cyto), E' = nuc+cyto

As above, we then compare these newly assigned term(s) with the true GO CC term(s) from dataset **D**.

In the COMMON variant, when a protein interacts with more than one other protein only those SCLs common to all its interaction partners are used to replace the masked terms. There are two possible cases:

1- if a protein interacts only with one other protein, the GO CC terms assigned to it come from the SCL annotated for that partner, as for the DISCRETE variant above; or

2- if a protein interacts with multiple partners, we assign the intersection of the partners' GO terms:

A' : B and A' : D; from B (nuc) and D (nuc+cyto), A' = nuc

The latter decision comes about because nuc is the only term common to the SCL annotations of both B and D.

MAJORITY differs in using only those GO CC terms present in annotations of at least half of the interacting partners of a protein. Using this method, assignments for B', D' and E' are identical to those in COMMON, but

A' : B and A' : D; from B (nuc) and D (nuc+cyto), A' = nuc+cyto

Although this outcome happens to be the same as for MERGED in this simple case, the reason is different (had an additional interaction A' : C (nuc) been present, we would infer A' = nuc under MAJORITY and A' = nuc+cyto under MERGED).

Obviously, further variants of arbitrary complexity could be developed, for example incorporating weighting of terms and/or based on machine-learning approaches or network analysis.

To evaluate these variants, we calculate accuracy from the counts of true (T) and false (F) predictions:

Accuracy = T/(T+F)

To penalise variants for potential over-prediction, we calculate both a permissive and a strict accuracy. For permissive accuracy (PA), a true prediction is scored for a protein if-and-only-if (iff) the true and predicted termsets have at least one term in common. A false prediction is scored iff no term from the prediction termset is found in the true termset. For strict accuracy (SA), a true prediction is scored iff the true and predicted termsets are identical; a false prediction is scored in all other cases. Our permissive calculations of true and false predictions are equivalent to the scoring scheme used previously to evaluate overall SCL prediction [2].

We evaluated the above four variants of our approach for each of four species using the reference datasets and subsets. Values for PA and SA are presented in Additional file 6. Over the datasets for human, mouse, fly and yeast the highest PA was often observed with the variant MERGED, while COMMON often demonstrated the highest SA. Here we discuss in detail only the results for human (Table 2); in the next section we evaluate our approach using the human union set and compare the results to the performance of four publicly available SCL prediction methods.

With human PPIs, PA values ranged from 0.64–0.85 for the DISCRETE variant of our approach, 0.72–0.86 for MERGED, 0.65–0.85 for COMMON, and 0.68–0.86 for MAJORITY (Table 2). For all variants, PA was higher in the subsets than in the reference dataset. The corresponding SA values fall between 0.35–0.58 for DISCRETE, 0.26–0.55 for MERGED, 0.40–0.57 for COMMON, and 0.34–0.56 for MAJORITY variants, and likewise are higher in subsets than in the parent reference set (Table 2). The COMMON variant usually performed as well as or better than the other variants, although at the cost of about 10% reduction in number of predictions, a consequence of the requirement that all protein interactors be annotated with at least one GO CC term in common.

Predicting the location of integral membrane proteins poses a particular challenge for sequence-based computational approaches [2]. We applied the MemO pipeline [36], which reports the consensus prediction among several methods, to identify and annotate transmembrane proteins in our various datasets, and used PPIs involving a predicted transmembrane protein as input into our four variant approaches. Overall, PA values for membrane proteins were slightly lower than for the whole reference set and subsets. PA values for transmembrane proteins ranged from 0.50–0.76 for the DISCRETE variant, 0.61–0.79 for MERGED, 0.53–0.77 for COMMON, 0.58–0.79 for MAJORITY (Table 2). For all variants, PA was higher in subsets than in the reference set except for PMID and DB subsets in the MERGED variant. As observed for PA, the SA values for membrane proteins are substantially lower as well (Table 2), indicating that, in general, predicting the SCL is more difficult for membrane proteins than for soluble proteins.

Returning to the issue of false predictions: it is important to appreciate that in some cases a pair of proteins that actually interact *in vivo *may correctly be annotated as located in different subcellular locations. This will be the case, for instance, for trans-membrane proteins that are embedded, in part, within a cellular membrane (*e.g*. the nuclear membrane) but possess one or more domains that extend into the adjacent compartment (*e.g*. the cytoplasm) and interact with a protein there. Our reference dataset contains 61 pairwise interactions between a Type I membrane protein and a soluble protein (see Additional file 7); many of the soluble proteins are annotated as located in the compartment (*e.g*. the cytoplasm) physically adjacent to the membrane in question (*e.g*. the nuclear membrane). These may (at least in principle) be true interactions, but according to the rules above are not identified as true positives. In the absence of a dependable, automated way to account for physical adjacency within the cell, the permissive and strict accuracy values reported for our approach may be too conservative.

The efficacy of our PPI-based approach depends on the quality of SCL annotation. The above results make it clear that PPI-based approaches have substantial potential as a predictor of SCL. Nonetheless significant issues remain, notably the tradeoff between quality and coverage.

### 9. Comparative evaluation of SCL prediction

We compared the performance of our SCL prediction methods (COMMON, MAJORITY and MERGED) to four publicly available SCL prediction methods, Proteome Analyst [52], WolfPSORT [53], CELLO [54] and pTARGET [55]. For this comparative evaluation we used our human union dataset ALL (Table 2), in which PPIs have at least one line of supporting evidence. PPIs in this set were divided into three groups according to the membrane organization of the interacting proteins: membrane-membrane, soluble-soluble, and membrane-soluble (mixed) interactions (see Methods, Section 4.1). Evaluation and comparison were conducted for each group. SCLs of all proteins in each group were predicted using our three methods (COMMON, MAJORITY and MERGED) and compared with the predictions of the publicly available prediction methods.

When applied to the PPI data involving only membrane proteins, all three variants were more accurate than any other SCL prediction methods (Table 3). Overall, our methods were 19–20 percentage points better than the best public method. On PPI data involving only soluble proteins, our variants performed as well as or better than the others (Table 3). The accuracy of our methods was 19–23 percentage points higher, except in comparison with Proteome Analyst.

**Table 3:** Evaluation of SCL prediction methods using human union set (ALL)

**PPI sets**	**COMMON**	**MAJORITY**	**MERGED**	**Proteome Analyst**	**WolfP** **SORT**	**CELLO**	**pTARGET**
**Membrane-membrane PPI only (162)**							
Total # of predicted proteins	155	162	162	35	162	162	79
# of correctly predicted proteins in PA	133	140	141	21	88	99	53
# of correctly predicted proteins in SA	92	91	84	9	61	56	38
PA	0.86	0.86	0.87	0.60	0.54	0.61	0.67
SA	0.59	0.56	0.52	0.26	0.38	0.35	0.48
							
**Soluble-soluble PPI only (807)**							
Total # of predicted proteins	686	803	807	411	807	807	499
# of correctly predicted proteins in PA	556	669	689	343	508	605	308
# of correctly predicted proteins in SA	350	372	314	211	320	367	193
PA	0.81	0.83	0.85	0.83	0.63	0.75	0.62
SA	0.51	0.46	0.39	0.51	0.40	0.45	0.39
							
**Membrane-soluble PPI only (206)**							
Total # of predicted proteins	193	205	206	64	206	206	95
# of correctly predicted proteins in PA	75	87	92	47	121	116	56
# of correctly predicted proteins in SA	39	37	35	32	92	75	39
PA	0.39	0.42	0.45	0.73	0.59	0.56	0.59
SA	0.20	0.18	0.17	0.50	0.45	0.36	0.41
							
**Total (1175)**							
Total # of predicted proteins	1034	1170	1175	510	1175	1175	673
# of correctly predicted proteins in PA	764	896	922	411	717	820	417
# of correctly predicted proteins in SA	481	500	433	252	473	498	270
PA	0.74	0.77	0.78	0.81	0.61	0.70	0.62
SA	0.47	0.43	0.37	0.49	0.40	0.42	0.40

* Numbers in the parentheses indicate total number of proteins for prediction for each category

In addition to the above sets, we constructed a dataset of interactions between transmembrane and soluble proteins. With these data, the accuracy of our methods was lower than the four public SCL prediction methods (Table 3) and lower than for the membrane-only and soluble-only datasets above. We suggest that this reduced performance is due to interactions among proteins from adjacent compartments; for example, one PPI in this group that generates incorrect predictions is an interaction between P31785 (IL2RG: cytokine receptor common gamma chain), localised to the plasma membrane, and its extracellular interaction partner, P13232 (IL7: interleukin-7), with an annotated location in the extracellular region [56]. Interactions such as these between proteins from adjacent compartments will confound our prediction methods, as seen in the results for this group. As mentioned previously (Section 8 and Additional file 7), this might be resolved through the combination of evidence such as the topology of interaction domains in each protein. As expected, we also observed better performance on the ALL subset than the reference set, and better performance on the BIO subset than on the EVI subset (see Table 3 and Additional file 8.1-3), reflecting the different proportions of co-PPIs previously observed in these subsets (Table 1).

When all PPIs are considered, our prediction methods performed better than three of the four public methods; Proteome Analyst showed somewhat higher PA and SA values (Table 3). Proteome Analyst retrieves SCL information by looking up fields of the Swiss-Prot knowledge base and returning a prediction [52]. Because our test data are derived from UniProt and therefore include Swiss-Prot annotations, it is expected that Proteome Analyst would perform well.

The existing methods were better at predicting the SCL of soluble proteins than of membrane proteins, but our methods showed markedly better performance when applied to interactions between membrane proteins. The methods that we evaluated in this study are based on the detection of features that determine SCL, either in protein sequences (WolfPSort, PTarget and Cello) or in protein knowledgebase records (ProteomeAnalyst). WolfPSORT, CELLO and pTARGET have largely been trained on soluble proteins, and this is reflected in their lower PA and SA values with membrane proteins. Proteome Analyst collects features from the SWISS-PROT KEYWORDS and SUBCELLULAR LOCATION fields of entries for proteins with the strongest BLAST matches to the query sequences [57]. If no protein is sufficiently similar, or if similar proteins have no features in those fields, Proteome Analyst does not return a prediction. We found few membrane proteins annotated with specific words in the SUBCELLULAR LOCATION field, and this likely explains the low coverage of membrane protein predictions by Proteome Analyst.

### 10. Validation on experimental data

As described above (Section 3.1), we applied our four variant approaches to infer GO CC term(s) for proteins of unknown SCL that interact with proteins of known SCL (potential prediction set: Additional file 1). Annotation of SCL is of course ongoing in the research community, and 72 mouse proteins (106 PPIs) without GO CC annotation at the time we carried out this inference subsequently received independent annotation based on experimentally determined SCL and were accessioned into the LOCSCL section of the LOCATE database [58]. We used these locations to validate predictions arising from our DISCRETE, MERGED, COMMON and MAJORITY variant approaches. After reassignment of GO CC terms as described above (Section 8), 106 SCL predictions were generated using DISCRETE, 72 using MERGED, 64 using COMMON and 72 using MAJORITY. Their PA and SA values are presented in Table 4.

**Table 4:** Evaluation of prediction method variants using LOCSCL

**PPI sets**	**DISCRETE**		**MERGED**		**COMMON**		**MAJORITY**	
	
	**PA**	**SA**	**PA**	**SA**	**PA**	**SA**	**PA**	**SA**
Reference	0.70	0.34	0.76	0.32	0.73	0.36	0.75	0.36
ALL	0.71	0.43	0.82	0.45	0.79	0.52	0.79	0.48
BIO	0.76	0.48	0.78	0.43	0.77	0.50	0.78	0.48
EVI	0.72	0.38	0.83	0.39	0.81	0.43	0.78	0.43

The four methods were applied to PPIs where one protein has a known SCL and the other SCL is unknown, generating new SCL predictions for the previously unlocated proteins. Permissive (PA) and strict accuracy (SA) were calculated based on the newly available SCL data for these proteins.

We observed that both accuracy values were essentially unchanged or slightly improved when compared with the reference set. The consistency of results across unknown as well as known data indicates that the detailed evaluation presented in Additional file 6 is likely to represent the actual performance of our variant methods on unknown data more generally. A similar validation can be constructed using the subset of the mouse reference set for which LOCATE annotations are available; results are presented in Additional file 9.

## Conclusion

This work was designed to explore the potential use of protein-protein interaction data in improving the prediction of subcellular localisation for eukaryotic proteins. We first evaluated the data contained in public PPI resources, and evaluated lines of evidence that reduce the potential for false positives in PPI data. We then developed four variations of an algorithm for inferring the localisation of proteins participating in PPIs, and evaluated these approaches against human data of known SCL and new experimental SCL data available for mouse proteins. Finally, we compared the performance of our methods with the performance of four publicly available SCL predictors, and demonstrated that our novel approach performs as well as or better.

To generate a comprehensive set of PPI data, we evaluated six publicly available PPI databases and constructed non-redundant PPI dataset from the data contained therein. We then collapsed and filtered GO terms for each protein to ensure consistent information and more-reliable evidence for location. We found that PPIs in human, mouse, fly and yeast tend to be recorded in only one database; thus combining data from multiple sources does in fact yield a more comprehensive coverage of known PPIs. We found that the three most-frequently annotated subcellular locations for proteins in known interactions are the nucleus, cytoplasm and plasma membrane, except in yeast where mitochondrion replaces plasma membrane in third position. This analysis also demonstrated the need for improved SCL annotation, as many protein annotations carry uncertain evidence codes. After filtration by evidence code and collapse of specific terms upward into parent terms in the GO hierarchy as described in Results and Discussion section 3.1, GO CC terms are available for 73% of the yeast proteins in our reference PPI datasets but for only 43% of mouse, 32% of human and 9% of fly proteins.

Concern is often expressed about the proportion of false positives in PPI data, particularly data generated using high-throughput techniques. We assumed, as a first approximation, that PPIs for which the two interacting proteins are located in different cellular compartments are false positive interactions (we return to reconsider this assumption later). Under this assumption, we showed that the co-PPI frequency in our reference set for each of the four species is 16–33 percentage points greater than random, i.e. substantial true-positive signal exists in our base data. We then demonstrated that the proportion of co-PPIs could be increased by applying, one at a time, six lines of additional supporting evidence describing the PPIs: co-PPI proportions were increased by 8–40 percentage points in 21 of the 24 subsets, and by up to 36 percentage points with the few combinations of evidence (union sets) that we tested. The greatest proportion of co-PPIs was always found in union sets supported by biological evidence type (Table 1). These improvements were counterbalanced to some extent, however, by decreased coverage of PPIs. We did not systematically explore further combinations of supporting evidence types. Scope appears to exist for the exploration of further lines of supporting evidence individually and, perhaps, in combination. Adaptive approaches based on machine learning would allow the combination of evidence types to be optimised for each dataset, although potentially at the expense of understanding specific features of the evidence in each case.

We developed four variants of an algorithmic approach that uses PPI data to infer protein location, and examined, singly and in simple combinations, the contribution of molecular (biological process, domain-domain interactions, interologs) and description-centric features (accession into multiple databases, citation of multiple literature items, support of multiple experimental detection methods) on the quality of prediction as judged by measures of permissive and strict accuracy. We evaluated our approach on human data (Table 2) and on a set of experimental SCL data recently generated for mouse proteins (Table 4), and demonstrated that MERGED has the highest PA, and COMMON the highest SA.

We compared the SCL prediction capacity of the COMMON, MAJORITY and MERGED methods to that of Proteome Analyst, WolfPSORT, CELLO and pTARGET; PA and SA values were examined for membrane-only, soluble-only, and mixed PPI subsets. Our methods always performed substantially better on the membrane-only subset, and usually better on the soluble-only subset. This demonstrates that considering membrane organization yields substantially better results in predicting SCL based on PPI data.

We also applied our approach to the mouse potential prediction set, inferring SCL for 783 proteins of unknown SCL. It transpired that SCL had recently been experimentally determined (independently of our work) and accessioned into a later version of the LOCATE database [58] for 72 proteins participating in 106 PPIs, allowing us to validate a subset of our predictions against these experimental data. We observed PA and SA values equal to or slightly higher than with the much larger reference dataset (see Additional file 9). Our MERGED method again yielded the best PA, while COMMON and MAJORITY tied for the best SA. We consider these results a validation of our approach, both in broad terms and in detail, and a strong indication that the performance against our reference sets is likely to be extensible to further eukaryotic (or at least animal) proteins more generally.

Additional directions remain to be explored. Multiple (non-pairwise) interactions and larger PPI networks may offer the possibility of inferring SCL based on secondary and tertiary as well as primary relationships. Challenges include those related to the reliability of PPI data, the quality of ontological annotation, tradeoffs between inference quality and coverage, adjacency of compartments and multi-molecular complexes within the cell, contingency of interaction, post-translational modification, and temporality of co-location. Our demonstration in this work that reliable PPI data can complement conventional experimental and computational approaches in identifying the SCL of proteins, provides a foundation on which these additional directions can be developed.

## Methods

### 1. Protein-protein interactions

#### 1.1 Protein-protein interaction datasets

We obtained protein-protein interaction (PPI) data for human, mouse, fly and yeast from six publicly available databases: Biomolecular Interaction Network Database (BIND: 25/06/2006) [59], Database of Interacting Proteins (DIP: 02/04/2006) [60], IntAct (07/07/2006) [61], Molecular INTeraction database (MINT: 05/2005) [62], MIPS Mammalian Protein-Protein Interaction database (MPPI) [63], and Human Protein Reference Database (HPRD:13/09/2005) [64].

#### 1.2 Standardization of identifiers

PPIs from different data sources were standardized by matching identifiers to UniProt accession numbers (ACs). GI numbers were mapped to UniProt ACs through PIR [15]. The previous version of UniProt ACs was also mapped to the most-recent version of UniProtKB release (v 12.1). In this standardization, the following cases were considered unmatched: (i) if identifiers match two different proteins within a species, and (ii) if identifiers converge into one accession number. In both cases, the corresponding proteins were removed.

Four of these databases (BIND, IntAct, DIP, MINT) contain protein from all four species. MPPI deals with only mammalian protein interactions, and HPRD only human protein interactions. In this study, we attempt to consider only pairwise PPIs; interactions within complexes were excluded, except (in the case of BIND) where further experimental evidence for direct pairwise physical interaction is cited. Standardization yielded 21121 non-redundant (NR) human PPIs (19686 heterodimers), 3032 NR mouse PPIs (2753 heterodimers), 31126 NR fly PPIs (30878 heterodimers) and 23586 NR yeast PPIs (22635 heterodimers). These constitute our in-house datasets.

#### 1.3 Reference sets

To obtain more-reliable annotation of the subcellular location (SCL) of these proteins, their associated GO terms (cellular component: CC) were filtered according to evidence codes provided by the Gene Ontology Consortium [65]. SCL annotation associated with the following codes was excluded: IC (Inferred by Curator), IEA (Inferred from Electronic Annotation), ISS (Inferred from Sequence or Structural Similarity), NAS (Non-traceable Author Statement), ND (No biological Data available), and NR (Not Recorded). For consistency, GO terms were collapsed from specific to general terms identifying the following 15 compartments: cytoplasm (GO:005737), cytoplasmic membrane-bound vesicle (GO:0016023), endoplasmic reticulum (GO:0005783), endosome (GO:0005768), extracellular matrix (GO:0031012), extracellular region (GO:0005576), Golgi apparatus (GO:0005794), lipid particle (GO:0005811), lysosome (GO:0005764), melanosome (GO:0042470), mitochondrion (GO:0005739), nucleus (GO:0005634), peroxisome (GO:0042470), plasma membrane (GO:0005886), and synaptic vesicle (GO:0008021).

After these consecutive processes, we obtained evidence-filtered, CC term-collapsed proteins: 2584 in human, 1062 in mouse, 784 in fly, and 3951 in yeast. These proteins take part in 3298 PPIs in human, 740 PPIs in mouse, 540 PPIs in fly and 16110 PPIs in yeast, in which both interacting proteins are distinct (i.e. the pairwise interaction is heterodimeric). We refer to these as our protein and PPI reference sets for each species.

#### 1.4 Subsets generated using additional lines of evidence

For each PPI in each of the four species-specific reference sets described immediately above, we looked for annotation in regard to six lines of supporting evidence, and on this basis constituted a total of 24 (4 species × 6 data types) subsets, each containing only those PPIs supported by one of these lines of evidence. The six lines of supporting evidence are:

i. interaction supported by similarity of GO Biological Process (BP) terms, as represented by the GO BP score [40];

ii. interaction supported by the presence of one or more domains known to participate in domain-domain interactions (DDIs) [41];

iii. interaction supported by presence of an equivalent interaction between a pair of orthologs (interolog) in another of these four species [42,43];

iv. interaction supported by presence in multiple databases;

v. interaction supported by literature; and

vi. interaction supported by experimental detection methods.

These lines of evidence are described more precisely in Results and Discussion, Section 6.1.

### 2. Subcellular location and Biological process information

#### 2.1 Subcellular location

The Gene Ontology (GO) Cellular Component (CC) annotation terms and evidence codes for each protein were obtained from UniProt Knowledgebase (version 12.1) to provide its SCL. To improve reliability, GO terms with less-reliable evidence were removed from each protein, after which GO terms remaining for each protein were collapsed into one of 15 defined locations (see Methods, Section 1.3). A few terms (*e.g*. nucleocytoplasmic shuttling complex) match to two different compartments and were collapsed into both high-level locations (for this example: nucleus, and cytoplasm).

#### 2.2 Biological process

To allow us to compare the biological process annotated for each of a pair of interacting proteins, we obtained the Biological Process ontology (BP) from UniProt (version 12.1) and recorded the evidence code for each BP term. BP terms were excluded according to the criteria applied to the CC terms (Methods, Section 1.3). Both proteins in a PPI were required to have at least one BP term each. Using these criteria, 6462 human PPIs (2950 proteins), 1136 mouse PPIs (1086 proteins), 1819 fly PPIs (1104 proteins), and 17087 yeast PPIs (3801 proteins) were available for analysis.

For each PPI, we used FSST to compute the average BPscore describing the similarity of GO BP terms and accepted only scores that equal or exceed the threshold 0.70 (see Additional file 2). Schlicker *et al*. [40] found that GO terms with BPscore 0.90 or greater were highly similar in function.

### 3. Interologs

Orthologs were obtained from the Inparanoid database [49]. If for an interacting pair in one species (*e.g*. human) each of the interacting proteins has an ortholog in another species (*e.g*. mouse) and that pair of orthologs interacts, then that second interacting pair (in this example, the pair in mouse) is referred to as the interolog of the interacting pair in human [47]. In the case of one-to-many co-orthology relationships, we considered all co-orthologs to contribute to interologs. (For example: the PPI A:B has been found in human. Protein A has ortholog A' in mouse, but B has co-orthologs B1' and B2'. We consider both A' :B1' and A' :B2' to be interologs of A:B. From the mouse perspective, A:B is the interolog of A' :B1' and equally the interolog of A' :B2').

### 4. Comparative evaluation of SCL prediction

#### 4.1 Input for SCL prediction

For comparative evaluation of SCL prediction, union set of all human PPIs where a PPI has at least one line of supporting evidence (ALL) was used. PPIs from ALL were categorized into three groups according to the membrane organization (MO) of interacting proteins in each PPI, resulting in three groups: 121 membrane-only PPI, involving 162 proteins, 938 soluble-only PPI, involving 807 proteins and 305 mixed PPI involving 206 proteins.

#### 4.2 Prediction of SCL

Prediction of SCL by three variants (COMMON, MAJORITY and MERGED) was carried out as illustrated in Section 8 of Results and Discussion. SCL of proteins in the membrane-only, soluble-only, and mixed PPI groups was assigned from interacting partners.

Proteins in each group were utilized as an input sequence to four publicly available and scalable SCL prediction methods: Proteome Analyst (PA-SUB 2.5) [52], WolfPSORT (last updated on 15/08/2007) [53], CELLO (v 2.5) [54] and pTARGET (last updated on 05/09/2006) [55,66]. We evaluated predictions against the annotated SCL in the union set. All public methods were run using the standard defaults for eukaryotic protein prediction.

In Proteome Analyst, proteins which were included in the training set of that method were not included. Because Proteome Analyst does not stipulate a threshold for positive prediction, we considered positive predictions to be those for which the associated probability was ≥ 90%. With WolfPSORT, we selected the prediction with the highest score. For CELLO, predictions with the highest probability were collected. If a method generated equal best predictions (i.e. two predictions with equal highest score or probability), both predictions were accepted.

Permissive and strict accuracy were examined for all predicted SCLs from three variants and four SCL prediction methods against SCLs of corresponding proteins in the ALL set.

### 5. Independent SCL data

We obtained SCL information for mouse proteins from the LOCATE database [58], a rigorously expert-curated resource based on independent empirical investigation and additional literature. In LOCATE, the SCL data generated by experimental investigation are clearly separated from SCL annotation extracted from the literature, and it is the experimental SCL data that we use in the validation presented here (Section 9). LOCATE utilizes an extension of the GO CC termset to describe additional or uncertain (*e.g*. membrane-like) SCLs. After matching identifiers in LOCATE to UniProt ACs and removing LOCATE-specific annotation terms, 1713 (of 2068 original) proteins and their locations (LOCSCL) remained. Of these, 83 proteins are found in the reference set, and 72 in the potential prediction set (Section 3.1). As LOCATE is not cited as literature for any protein in UniProt, we expect the SCL information in LOCATE to be independent of UniProt annotation.

### 6. Consistent annotation of detection method

PPI datasets from the resources used here (Methods, Section 1) are frequently annotated with a description of the method used to detect the interaction. These terms are defined in the Protein Standards Initiative (PSI) Molecular Interaction (MI) ontology. It is known that PPI database curators sometimes annotate a single detection method recorded in a given paper using different terms [67], so we collapsed method annotations from the PSI-MI Experimental Interaction Detection Method (MI:0045) hierarchy of the PSI-MI ontology upward into the four main high-level categories of this term: Biophysical assay (MI:0013), Protein complementation assay (MI:0090), Biochemical assay (MI:0401), and Imaging techniques (MI:0428). Other, less-frequently cited experimental methods that did not collapse to these four high-level terms were grouped as "Others". As PSI-MI permits multiple inheritance, any term (*e.g*. Bacterial display, MI:0009) which mapped to two or more high-level categories was placed into the "Others" category. These data were used to examine the distribution of experimental methods for detecting PPIs across PPI databases (Results and Discussion, Section 2.3) and using detection method as a line of supporting evidence (Results and Discussion, Section 6.1-vi).

## Availability and requirements

Our protein and PPI datasets for the four species are available at  (under Tools and Data/Databases and Datasets), from the link "PPI – Shin et al. (2009)". Perl scripts implementing the four variants of our prediction method are linked from the same page.

## Abbreviations

BIO: subset constituted by union of biological evidence types; BPscore: Biological Process score; CC: Cellular Component (hierarchy within Gene Ontology); DDI: domain-domain interaction; EVI: subset constituted by union of recorded evidence types; GO: Gene Ontology; PA: permissive accuracy; PPI: protein-protein interaction; SA: strict accuracy; SCL: subcellular location.

## Authors' contributions

CJS conducted all experiments and analysed the data. CJS and SW designed the experiments. MJD and CJS designed and evaluated the SCL inference methods which were implemented by CJS. MJD assisted with biological interpretation. MAR supervised the project. All authors contributed to writing the manuscript.

## Supplementary Material

Additional file 1 **The number of PPIs according to presence of GO CC for protein and PPI.** In heterodimeric PPIs, the two interacting proteins are different. The reference set for this study consists of all PPIs in which a GO CC term is available for each of the two interacting proteins. We identify a further set of PPIs for which one protein in each interacting pair has a GO CC term and the other one does not; this constitutes our potential prediction set. The remaining PPIs have no associated GO CC terms and are not informative for this study. Click here for file

Additional file 2 **Coverage and proportion of PPIs.** These data are drawn from the subsets supported by the *BPscore *line of evidence. Click here for file

Additional file 3 **Comparison of protein SCL between interologs.** The SCL of proteins in human PPIs was compared with location of their orthologs from mouse, fly and yeast interologs. Click here for file

Additional file 4 **PPI subsets based on low-throughput experimental data.** DB, Method and PMID subsets were generated as described in Methods section 1.4, based on a reference set consisting only of those PPIs detected by low-throughput experimental methods. Click here for file

Additional file 5 **Numbers of PPIs annotated with PSI-MI experimental detection methods.** These data are drawn from the subsets supported by the *experimental detection methods *line of evidence, for the four species under consideration. Click here for file

Additional file 6 **Evaluation of prediction method variants on reference and supported subsets for human, mouse, fly and yeast.** The permissive accuracy (PA) and strict accuracy (SA) were calculated for all variants (DISCRETE, MERGED, COMMON and MAJORITY) for all interactions, and for interactions involving an integral membrane protein (M). Click here for file

Additional file 7 **Interactions between Type I integral membrane proteins and soluble intracellular proteins in human.** The PPIs in adjacent compartments (Type I integral membrane proteins and soluble intracellular proteins) are listed for human; only domains present as interacting pairs in the iPfam subset are included. Click here for file

Additional file 8 **Evaluation of SCL prediction methods using human reference set and subsets.** SCL prediction methods, including three variants of our approach (COMMON, MAJORITY, MERGED) and four existing methods were compared using the human reference set (REF) and two different unions of subsets (BIO and EVI). Click here for file

Additional file 9 **Evaluation of prediction method variants using LOCSCL.** These 146 PPIs come from the mouse reference set and have additional SCL annotation data available from LOCATE [43]. They are here evaluated against LOCATE SCL annotations. Where both interaction partners have LOCSCL data, the randomization process was applied. Click here for file
