# Supplementary material for: Protein-protein interaction as a predictor of subcellular location
Source: BMC Syst Biol. 2009 Feb 25;3:28. doi: 10.1186/1752-0509-3-28 (PMC2663780; doi:10.1186/1752-0509-3-28)
Supplement: Additional file 1 — The number of PPIs according to presence of GO CC for protein and PPI. In heterodimeric PPIs, the two interacting proteins are different. The reference set for this study consists of all PPIs in which a GO CC term is available for each of the two interacting proteins. We identify a further set of PPIs for which one protein in each interacting pair has a GO CC term and the other one does not; this constitutes our potential prediction set. The remaining PPIs have no associated GO CC terms and are not informative for this study. [file 1752-0509-3-28-S1.pdf]

Additional file 1 - The number of PPIs according to presence of GO CC for protein and PPI

In heterodimeric PPIs, the two interacting proteins are different. The reference set for this study consists of all PPIs in which a GO CC term is available for each of the two interacting proteins. We identify a further set of PPIs for which one protein in each interacting pair has a GO CC term and the other one does not; this constitutes our potential prediction set. The remaining PPIs have no associated GO CC terms and are not informative for this study.

PPI sets	Human			Mouse			Fly			Yeast		
	# PPIs	# proteins in PPI set with GO	# proteins in PPI set without GO	# PPIs	# proteins in PPI set with GO	# proteins in PPI set without GO	# PPIs	# proteins in PPI set with GO	# proteins in PPI set without GO	# PPIs	# proteins in PPI set with GO	# proteins in PPI set without GO
Total # of heterodimeric PPIs	19686	2494	5399	2753	1029	1359	30878	782	7996	22635	3938	1488
Reference set	3298	1860	--	740	780	--	540	461	--	16110	3837	--
Potential prediction set	8830	2083	3280	1239	587	783	5775	716	2998	5727	1999	1406
# of PPIs with no known SCL	7558	--	4364	774	--	876	24563	--	7567	798	--	663
