# Supplementary material for: Protein-protein interaction as a predictor of subcellular location
Source: BMC Syst Biol. 2009 Feb 25;3:28. doi: 10.1186/1752-0509-3-28 (PMC2663780; doi:10.1186/1752-0509-3-28)

Additional file 2 - Coverage and proportion of PPIs

These data are drawn from the subsets supported by the *BPscore* line of evidence. Panels a-d show the proportion of human, mouse, fly and yeast reference PPIs supported (coverage), and the proportion of co-PPIs, for thresholds from 0.1 to 0.9. Blue circles, coverage; red circles, co-PPIs.

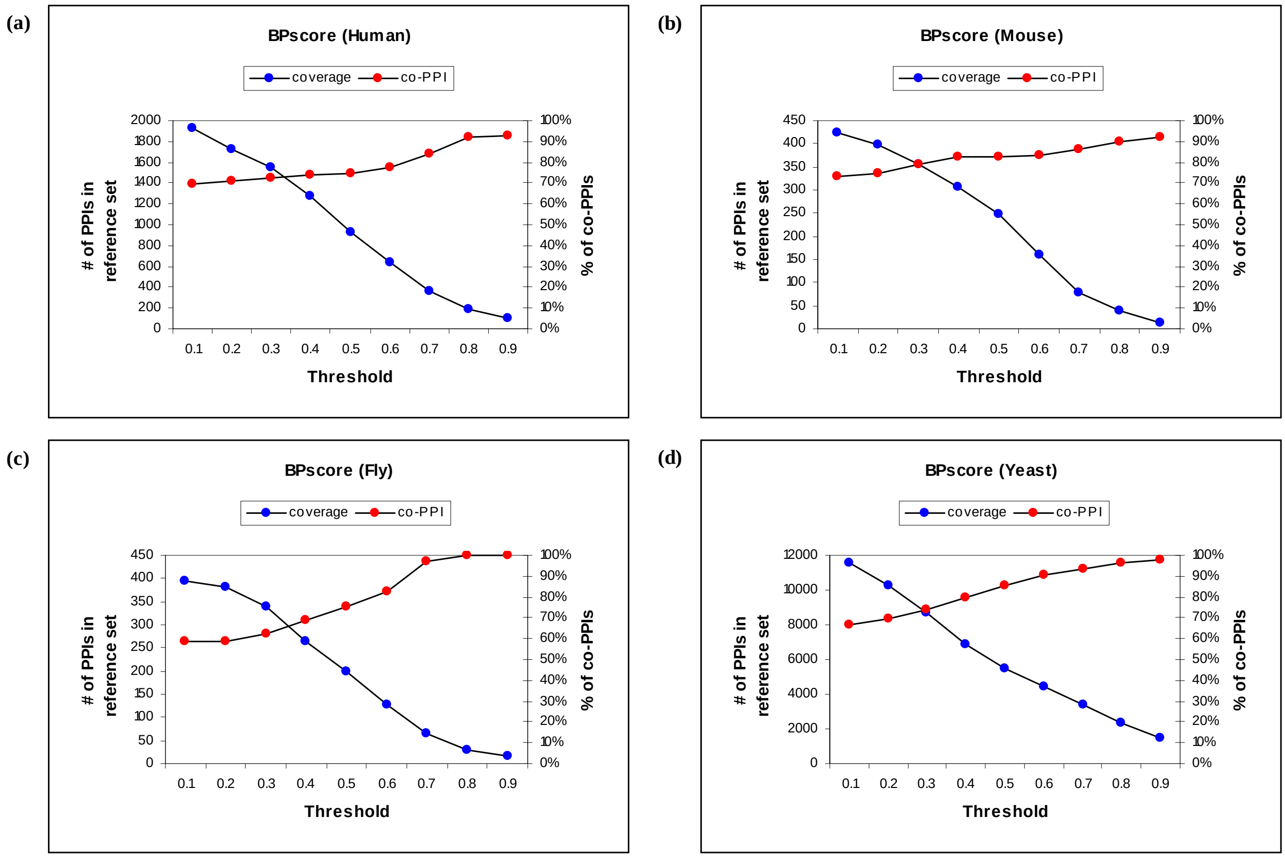

Supplement: Additional file 2 — Coverage and proportion of PPIs. These data are drawn from the subsets supported by the BPscore line of evidence. [file 1752-0509-3-28-S2.pdf]
