# Supplementary material for: Protein-protein interaction as a predictor of subcellular location
Source: BMC Syst Biol. 2009 Feb 25;3:28. doi: 10.1186/1752-0509-3-28 (PMC2663780; doi:10.1186/1752-0509-3-28)
Supplement: Additional file 3 — Comparison of protein SCL between interologs. The SCL of proteins in human PPIs was compared with location of their orthologs from mouse, fly and yeast interologs. [file 1752-0509-3-28-S3.pdf]

Additional file 3 - Comparison of protein SCL between interologs

The SCL of proteins in human PPIs was compared with location of their orthologs from mouse, fly and yeast interologs. Shaded rows indicate cases where these locations differ. PM, plasma membrane; CMV, cytoplasmic membrane bounded-vesicle; ER, endoplasmic reticulum.

Species	Protein A	Protein B	GO CC	GO CC	Location A	Location B	Species	Protein C	Protein D	GO CC	GO CC	Location C	Location D
Human	P14598	Q15080	GO:0005737	GO:0005737<br>GO:0005886	Cytoplasm	Cytoplasm<br>PM	Mouse	P97369	Q09014	GO:0005737	GO:0005737	Cytoplasm	Cytoplasm
Human	P51636	Q03135	GO:0005886<br>GO:0005737<br>GO:0016023	GO:0005886<br>GO:0005783<br>GO:0005794	PM<br>Cytoplasm<br>CMV	PM<br>ER<br>Golgi	Mouse	P49817	Q9WVC3	GO:0005886<br>GO:0005737	GO:0005886	PM<br>Cytoplasm	PM
Human	P01100	P05412	GO:0005634	GO:0005634	Nucleus	Nucleus	Mouse	P01101	P05627	GO:0005634	GO:0005634	Nucleus	Nucleus
Human	P06400	P17676	GO:0005634	GO:0005634	Nucleus	Nucleus	Mouse	P13405	P28033	GO:0005634	GO:0005634<br>GO:0005737	Nucleus	Nucleus<br>Cytoplasm
Human	P49736	Q13416	GO:0005634	GO:0005634	Nucleus	Nucleus	Mouse	P97310	Q60862	GO:0005634	GO:0005634	Nucleus	Nucleus
Human	P33993	P49736	GO:0005634	GO:0005634	Nucleus	Nucleus	Mouse	P97310	Q61881	GO:0005634	GO:0005634	Nucleus	Nucleus
Human	Q13415	Q13416	GO:0005634	GO:0005634	Nucleus	Nucleus	Mouse	Q60862	Q9Z1N2	GO:0005634	GO:0005634	Nucleus	Nucleus
Human	P19838	Q13547	GO:0005634<br>GO:0005737	GO:0005634<br>GO:0005737	Nucleus<br>Cytoplasm	Nucleus<br>Cytoplasm	Mouse	O09106	P25799	GO:0005634<br>GO:0005737	GO:0005634	Nucleus<br>Cytoplasm	Nucleus
Human	Q04206	Q13547	GO:0005634<br>GO:0005737	GO:0005634<br>GO:0005737	Nucleus<br>Cytoplasm	Nucleus<br>Cytoplasm	Mouse	O09106	Q04207	GO:0005634<br>GO:0005737	GO:0005634<br>GO:0005737	Nucleus<br>Cytoplasm	Nucleus<br>Cytoplasm
Human	P19838	Q04206	GO:0005634<br>GO:0005737	GO:0005634<br>GO:0005737	Nucleus<br>Cytoplasm	Nucleus<br>Cytoplasm	Mouse	P25799	Q04207	GO:0005634	GO:0005634<br>GO:0005737	Nucleus	Nucleus<br>Cytoplasm
Human	P25963	Q04206	GO:0005634<br>GO:0005737	GO:0005634<br>GO:0005737	Nucleus<br>Cytoplasm	Nucleus<br>Cytoplasm	Mouse	Q04207	Q9Z1E3	GO:0005634<br>GO:0005737	GO:0005634<br>GO:0005737	Nucleus<br>Cytoplasm	Nucleus<br>Cytoplasm
Human	Q9UQL6	Q9Y618	GO:0005634<br>GO:0005737	GO:0005634	Nucleus<br>Cytoplasm	Nucleus	Mouse	Q9WU42	Q9Z2V6	GO:0005634	GO:0005634<br>GO:0005737	Nucleus	Nucleus<br>Cytoplasm
Human	Q07157	Q16625	GO:0005886	GO:0005886	PM	PM	Mouse	P39447	Q61146	GO:0005886<br>GO:0005634<br>GO:0005737	GO:0005886	PM<br>Nucleus<br>Cytoplasm	PM
Human	P33151	P35222	GO:0005886	GO:0005886<br>GO:0005634	PM	PM<br>Nucleus	Mouse	P55284	Q02248	GO:0005886	GO:0005886<br>GO:0005634<br>GO:0005737	PM	PM<br>Nucleus<br>Cytoplasm
Human	Q13416	Q99741	GO:0005634	GO:0005634<br>GO:0005737	Nucleus	Nucleus<br>Cytoplasm	Mouse	O89033	Q60862	GO:0005737	GO:0005634	Cytoplasm	Nucleus
Human	P33993	Q99741	GO:0005634	GO:0005634<br>GO:0005737	Nucleus	Nucleus<br>Cytoplasm	Mouse	O89033	Q61881	GO:0005737	GO:0005634	Cytoplasm	Nucleus
Human	Q13415	Q99741	GO:0005634	GO:0005634<br>GO:0005737	Nucleus	Nucleus<br>Cytoplasm	Mouse	O89033	Q9Z1N2	GO:0005737	GO:0005634	Cytoplasm	Nucleus

Human	P10415	Q99933	GO:0005634<br>GO:0005739	GO:0005634<br>GO:0005737	Nucleus<br>Mitochondrion	Nucleus<br>Cytoplasm	Mouse	P10417	Q60739	GO:0005737<br>GO:0005739	GO:0005634<br>GO:0005737	Cytoplasm<br>Mitochondrion	Nucleus<br>Cytoplasm
Human	P98161	Q13563	GO:0005886	GO:0005737<br>GO:0005886	PM	Cytoplasm<br>PM	Mouse	O08852	O35245	GO:0005634<br>GO:0005886	GO:0005783	Nucleus<br>PM	ER
Human	O60563	P50750	GO:0005634	GO:0005634	Nucleus	Nucleus	Fly	O17432	O96433	GO:0005634	GO:0005634	Nucleus	Nucleus
Human	P11387	Q9NS56	GO:0005634	GO:0005634	Nucleus	Nucleus	Fly	P30189	Q9V8P9	GO:0005634<br>GO:0005737	GO:0005634	Nucleus<br>Cytoplasm	Nucleus
Human	P26368	Q01081	GO:0005634	GO:0005634	Nucleus	Nucleus	Fly	Q24562	Q94535	GO:0005634	GO:0005634	Nucleus	Nucleus
Human	P25963	Q04206	GO:0005737<br>GO:0005634	GO:0005737<br>GO:0005634	Cytoplasm<br>Nucleus	Cytoplasm<br>Nucleus	Fly	P15330	Q03017	GO:0005737<br>GO:0005634	GO:0005737	Cytoplasm<br>Nucleus	Cytoplasm
Human	Q15528	Q9NVC6	GO:0005634<br>GO:0005737	GO:0005634	Nucleus<br>Cytoplasm	Nucleus	Fly	Q9V439	Q9VEC1	GO:0005634	GO:0005634	Nucleus	Nucleus
Human	P25054	P35222	GO:0005886<br>GO:0005634<br>GO:0005737	GO:0005886<br>GO:0005634	PM<br>Nucleus<br>Cytoplasm	PM<br>Nucleus	Fly	P18824	Q9Y1T2	GO:0005886<br>GO:0005737	GO:0005886	PM<br>Cytoplasm	PM
Human	P10071	Q9UMX1	GO:0005634<br>GO:0005737	GO:0005634	Nucleus<br>Cytoplasm	Nucleus	Fly	P19538	Q27279	GO:0005634<br>GO:0005737	GO:0005737	Nucleus<br>Cytoplasm	Cytoplasm
Human	P05386	P05387	GO:0005737	GO:0005737	Cytoplasm	Cytoplasm	Yeast	P02400	P10622	GO:0005737	GO:0005737	Cytoplasm	Cytoplasm
Human	P11940	Q04637	GO:0005737	GO:0005737	Cytoplasm	Cytoplasm	Yeast	P04147	P39935	GO:0005737<br>GO:0005634	GO:0005737<br>GO:0005739	Cytoplasm<br>Nucleus	Cytoplasm<br>Mitochondrion
Human	O43432	P11940	GO:0005737	GO:0005737	Cytoplasm	Cytoplasm	Yeast	P04147	P39935	GO:0005737<br>GO:0005634	GO:0005737<br>GO:0005739	Cytoplasm<br>Nucleus	Cytoplasm<br>Mitochondrion
Human	P11940	Q04637	GO:0005737	GO:0005737	Cytoplasm	Cytoplasm	Yeast	P04147	P39936	GO:0005737<br>GO:0005634	GO:0005737	Cytoplasm<br>Nucleus	Cytoplasm
Human	O43432	P11940	GO:0005737	GO:0005737	Cytoplasm	Cytoplasm	Yeast	P04147	P39936	GO:0005737<br>GO:0005634	GO:0005737	Cytoplasm<br>Nucleus	Cytoplasm
Human	P55884	Q14152	GO:0005737	GO:0005737	Cytoplasm	Cytoplasm	Yeast	P06103	P38249	GO:0005737	GO:0005737	Cytoplasm	Cytoplasm
Human	P06730	Q04637	GO:0005737	GO:0005737	Cytoplasm	Cytoplasm	Yeast	P07260	P39935	GO:0005737<br>GO:0005634	GO:0005737<br>GO:0005739	Cytoplasm<br>Nucleus	Cytoplasm<br>Mitochondrion
Human	O43432	P06730	GO:0005737	GO:0005737	Cytoplasm	Cytoplasm	Yeast	P07260	P39935	GO:0005737<br>GO:0005634	GO:0005737<br>GO:0005739	Cytoplasm<br>Nucleus	Cytoplasm<br>Mitochondrion
Human	P06730	Q04637	GO:0005737	GO:0005737	Cytoplasm	Cytoplasm	Yeast	P07260	P39936	GO:0005737<br>GO:0005634	GO:0005737	Cytoplasm<br>Nucleus	Cytoplasm
Human	O43432	P06730	GO:0005737	GO:0005737	Cytoplasm	Cytoplasm	Yeast	P07260	P39936	GO:0005737<br>GO:0005634	GO:0005737	Cytoplasm<br>Nucleus	Cytoplasm
Human	P08237	P17858	GO:0005737	GO:0005737	Cytoplasm	Cytoplasm	Yeast	P16861	P16862	GO:0005737<br>GO:0005739	GO:0005737<br>GO:0005739	Cytoplasm<br>Mitochondrion	Cytoplasm<br>Mitochondrion
Human	P08237	P17858	GO:0005737	GO:0005737	Cytoplasm	Cytoplasm	Yeast	P16861	P16862	GO:0005737<br>GO:0005739	GO:0005737<br>GO:0005739	Cytoplasm<br>Mitochondrion	Cytoplasm<br>Mitochondrion
Human	Q99442	Q9UGP8	GO:0005783	GO:0005783	ER	ER	Yeast	P14906	P21825	GO:0005783<br>GO:0005739	GO:0005783	ER<br>Mitochondrion	ER
Human	O43292	Q969N2	GO:0005783	GO:0005783	ER	ER	Yeast	P38875	P39012	GO:0005783	GO:0005783	ER	ER
Human	Q92643	Q969N2	GO:0005783	GO:0005783	ER	ER	Yeast	P38875	P49018	GO:0005783	GO:0005783	ER	ER

Human	O43292	Q92643	GO:0005783	GO:0005783	ER	ER	Yeast	P39012	P49018	GO:0005783	GO:0005783	ER	ER
Human	P11387	P19338	GO:0005634	GO:0005634<br>GO:0005737	Nucleus	Nucleus<br>Cytoplasm	Yeast	P04786	P27476	GO:0005634	GO:0005634<br>GO:0005739	Nucleus	Nucleus<br>Mitochondrion
Human	P27694	P43351	GO:0005634	GO:0005634	Nucleus	Nucleus	Yeast	P06778	P22336	GO:0005634	GO:0005634<br>GO:0005737	Nucleus	Nucleus<br>Cytoplasm
Human	P15927	P43351	GO:0005634	GO:0005634	Nucleus	Nucleus	Yeast	P06778	P26754	GO:0005634	GO:0005634	Nucleus	Nucleus
Human	P18074	P19447	GO:0005634	GO:0005634	Nucleus	Nucleus	Yeast	P06839	Q00578	GO:0005634	GO:0005634	Nucleus	Nucleus
Human	P49736	Q99741	GO:0005634	GO:0005634<br>GO:0005737	Nucleus	Nucleus<br>Cytoplasm	Yeast	P09119	P29469	GO:0005634	GO:0005634<br>GO:0005737	Nucleus	Nucleus<br>Cytoplasm
Human	Q13416	Q99741	GO:0005634	GO:0005634<br>GO:0005737	Nucleus	Nucleus<br>Cytoplasm	Yeast	P09119	P32833	GO:0005634	GO:0005634	Nucleus	Nucleus
Human	O43913	Q99741	GO:0005634	GO:0005634<br>GO:0005737	Nucleus	Nucleus<br>Cytoplasm	Yeast	P09119	P50874	GO:0005634	GO:0005634	Nucleus	Nucleus
Human	P49959	Q92878	GO:0005634	GO:0005634	Nucleus	Nucleus	Yeast	P12753	P32829	GO:0005634<br>GO:0005739	GO:0005634<br>GO:0005739	Nucleus<br>Mitochondrion	Nucleus<br>Mitochondrion
Human	P09884	Q07864	GO:0005634	GO:0005634	Nucleus	Nucleus	Yeast	P13382	P21951	GO:0005634<br>GO:0005739	GO:0005634	Nucleus<br>Mitochondrion	Nucleus
Human	O15514	P19388	GO:0005634	GO:0005634	Nucleus	Nucleus	Yeast	P20433	P20434	GO:0005634<br>GO:0005737	GO:0005634	Nucleus<br>Cytoplasm	Nucleus
Human	P18754	P43487	GO:0005634	GO:0005634<br>GO:0005737	Nucleus	Nucleus<br>Cytoplasm	Yeast	P21827	P41920	GO:0005634	GO:0005634<br>GO:0005737	Nucleus	Nucleus<br>Cytoplasm
Human	P15927	P27694	GO:0005634	GO:0005634	Nucleus	Nucleus	Yeast	P22336	P26754	GO:0005634<br>GO:0005737	GO:0005634	Nucleus<br>Cytoplasm	Nucleus
Human	P25205	P49736	GO:0005634	GO:0005634	Nucleus	Nucleus	Yeast	P24279	P29469	GO:0005634<br>GO:0005737	GO:0005634<br>GO:0005737	Nucleus<br>Cytoplasm	Nucleus<br>Cytoplasm
Human	P25205	P33992	GO:0005634	GO:0005634	Nucleus	Nucleus	Yeast	P24279	P29496	GO:0005634<br>GO:0005737	GO:0005634<br>GO:0005737	Nucleus<br>Cytoplasm	Nucleus<br>Cytoplasm
Human	P25205	P33993	GO:0005634	GO:0005634	Nucleus	Nucleus	Yeast	P24279	P38132	GO:0005634<br>GO:0005737	GO:0005634<br>GO:0005737	Nucleus<br>Cytoplasm	Nucleus<br>Cytoplasm
Human	P15927	P23025	GO:0005634	GO:0005634	Nucleus	Nucleus	Yeast	P26754	P28519	GO:0005634	GO:0005634	Nucleus	Nucleus
Human	P33992	P49736	GO:0005634	GO:0005634	Nucleus	Nucleus	Yeast	P29469	P29496	GO:0005634<br>GO:0005737	GO:0005634<br>GO:0005737	Nucleus<br>Cytoplasm	Nucleus<br>Cytoplasm
Human	P33992	P33993	GO:0005634	GO:0005634	Nucleus	Nucleus	Yeast	P29496	P38132	GO:0005634<br>GO:0005737	GO:0005634<br>GO:0005737	Nucleus<br>Cytoplasm	Nucleus<br>Cytoplasm
Human	O43913	Q13416	GO:0005634	GO:0005634	Nucleus	Nucleus	Yeast	P32833	P50874	GO:0005634	GO:0005634	Nucleus	Nucleus
Human	O43929	Q13416	GO:0005634	GO:0005634	Nucleus	Nucleus	Yeast	P32833	P54791	GO:0005634	GO:0005634	Nucleus	Nucleus
Human	O14777	Q14683	GO:0005634	GO:0005634	Nucleus	Nucleus	Yeast	P32908	P40460	GO:0005634	GO:0005634	Nucleus	Nucleus
Human	P49848	Q15542	GO:0005634	GO:0005634	Nucleus	Nucleus	Yeast	P38129	P53040	GO:0005634	GO:0005634	Nucleus	Nucleus
Human	O75419	P33993	GO:0005634	GO:0005634	Nucleus	Nucleus	Yeast	P38132	Q08032	GO:0005634<br>GO:0005737	GO:0005634	Nucleus<br>Cytoplasm	Nucleus
Human	P35250	P35251	GO:0005634	GO:0005634	Nucleus	Nucleus	Yeast	P38630	P40339	GO:0005634	GO:0005634	Nucleus	Nucleus
Human	O43913	O43929	GO:0005634	GO:0005634	Nucleus	Nucleus	Yeast	P50874	P54791	GO:0005634	GO:0005634	Nucleus	Nucleus

Human	Q13435	Q15393	GO:0005634	GO:0005634	Nucleus	Nucleus	Yeast	Q02554	Q04693	GO:0005634	GO:0005634	Nucleus	Nucleus
Human	Q13435	Q15427	GO:0005634	GO:0005634	Nucleus	Nucleus	Yeast	Q02554	Q99181	GO:0005634	GO:0005634	Nucleus	Nucleus
Human	P55854	Q8N2W9	GO:0005634	GO:0005634	Nucleus	Nucleus	Yeast	Q12216	Q12306	GO:0005634<br>GO:0005737	GO:0005634<br>GO:0005737	Nucleus<br>Cytoplasm	Nucleus<br>Cytoplasm
Human	P24941	P25205	GO:0005634<br>GO:0005737	GO:0005634	Nucleus<br>Cytoplasm	Nucleus	Yeast	P00546	P24279	GO:0005634<br>GO:0005737	GO:0005634<br>GO:0005737	Nucleus<br>Cytoplasm	Nucleus<br>Cytoplasm
Human	P24941	Q13416	GO:0005634<br>GO:0005737	GO:0005634	Nucleus<br>Cytoplasm	Nucleus	Yeast	P00546	P32833	GO:0005634<br>GO:0005737	GO:0005634	Nucleus<br>Cytoplasm	Nucleus
Human	O00311	P49736	GO:0005634<br>GO:0005737	GO:0005634	Nucleus<br>Cytoplasm	Nucleus	Yeast	P06243	P29469	GO:0005634	GO:0005634<br>GO:0005737	Nucleus	Nucleus<br>Cytoplasm
Human	O00410	P49790	GO:0005634<br>GO:0005737	GO:0005634	Nucleus<br>Cytoplasm	Nucleus	Yeast	P14907	P32337	GO:0005634	GO:0005634<br>GO:0005737	Nucleus	Nucleus<br>Cytoplasm
Human	Q15019	Q9UHD8	GO:0005737<br>GO:0005634	GO:0005737	Cytoplasm<br>Nucleus	Cytoplasm	Yeast	P25342	P32468	GO:0005737	GO:0005737	Cytoplasm	Cytoplasm
Human	O14980	Q9H6Z4	GO:0005634<br>GO:0005737	GO:0005634	Nucleus<br>Cytoplasm	Nucleus	Yeast	P30822	P40517	GO:0005634	GO:0005634	Nucleus	Nucleus
Human	Q7L523	Q9HB90	GO:0005634<br>GO:0005737	GO:0005634<br>GO:0005737	Nucleus<br>Cytoplasm	Nucleus<br>Cytoplasm	Yeast	P53290	Q00582	GO:0005634<br>GO:0005737<br>GO:0005768	GO:0005634<br>GO:0005737<br>GO:0005768	Nucleus<br>Cytoplasm<br>Endosome	Nucleus<br>Cytoplasm<br>Endosome
Human	Q9Y230	Q9Y265	GO:0005634<br>GO:0005737	GO:0005634	Nucleus<br>Cytoplasm	Nucleus	Yeast	Q03940	Q12464	GO:0005634	GO:0005634	Nucleus	Nucleus
Human	O75381	Q92968	GO:0005777	GO:0005777	Peroxisome	Peroxisome	Yeast	P53112	P80667	GO:0005777	GO:0005777	Peroxisome	Peroxisome
Human	P30260	Q13042	GO:0005737	GO:0005737	Cytoplasm	Cytoplasm	Yeast	P09798	P38042	GO:0005634	GO:0005634	Nucleus	Nucleus
Human	Q92900	Q9HAU5	GO:0005634	GO:0005634<br>GO:0005737	Nucleus	Nucleus<br>Cytoplasm	Yeast	P30771	P38798	GO:0005737	GO:0005737	Cytoplasm	Cytoplasm
Human	O15116	Q9Y4Z0	GO:0005634	GO:0005634	Nucleus	Nucleus	Yeast	P40070	P47017	GO:0005634	GO:0005737	Nucleus	Cytoplasm
Human	P08107	P31948	GO:0005634<br>GO:0005739<br>GO:0005783	GO:0005634<br>GO:0005794	Nucleus<br>Mitochondrion<br>ER	Nucleus<br>Golgi	Yeast	P09435	P15705	GO:0005737	GO:0005737	Cytoplasm	Cytoplasm
Human	P08107	P31948	GO:0005634<br>GO:0005739<br>GO:0005783	GO:0005634<br>GO:0005794	Nucleus<br>Mitochondrion<br>ER	Nucleus<br>Golgi	Yeast	P10592	P15705	GO:0005737<br>GO:0005739	GO:0005737	Cytoplasm<br>Mitochondrion	Cytoplasm
Human	P08107	P31948	GO:0005634<br>GO:0005739<br>GO:0005783	GO:0005634<br>GO:0005794	Nucleus<br>Mitochondrion<br>ER	Nucleus<br>Golgi	Yeast	P15705	P22202	GO:0005737	GO:0005634<br>GO:0005737	Cytoplasm	Nucleus<br>Cytoplasm
