# Supplementary material for: Protein-protein interaction as a predictor of subcellular location
Source: BMC Syst Biol. 2009 Feb 25;3:28. doi: 10.1186/1752-0509-3-28 (PMC2663780; doi:10.1186/1752-0509-3-28)
Supplement: Additional file 4 — PPI subsets based on low-throughput experimental data. DB, Method and PMID subsets were generated as described in Methods section 1.4, based on a reference set consisting only of those PPIs detected by low-throughput experimental methods. [file 1752-0509-3-28-S4.pdf]

Additional file 4 – PPI subsets based on low-throughput experimental data

DB, Method and PMID subsets were generated as described in Methods section 1.4, based on a reference set consisting only of those PPIs detected by low-throughput experimental methods.

PPI	Human			Mouse			Fly			Yeast		
	**Total PPI (#)**	**Co-PPI (#)**	**Co-PPI (%)**	**Total PPI (#)**	**Co-PPI (#)**	**Co-PPI (%)**	**Total PPI (#)**	**Co-PPI (#)**	**Co-PPI (%)**	**Total PPI (#)**	**Co-PPI (#)**	**Co-PPI (%)**
DB	317 (10%)	229	72%	40 (5%)	36	90%	4 (1%)	4	100%	402 (2%)	344	86%
Method	98 (3%)	73	74%	27 (4%)	23	85%	1	1	100%	235 (1%)	218	93%
PMID	83 (3%)	58	70%	16 (2%)	13	81%	7 (1%)	6	86%	568 (4%)	513	90%
