# Supplementary material for: Protein-protein interaction as a predictor of subcellular location
Source: BMC Syst Biol. 2009 Feb 25;3:28. doi: 10.1186/1752-0509-3-28 (PMC2663780; doi:10.1186/1752-0509-3-28)
Supplement: Additional file 5 — Numbers of PPIs annotated with PSI-MI experimental detection methods. These data are drawn from the subsets supported by the experimental detection methods line of evidence, for the four species under consideration. [file 1752-0509-3-28-S5.pdf]

Additional file 5 – Numbers of PPIs annotated with PSI-MI experimental detection methods

These data are drawn from the subsets supported by the *experimental detection methods* line of evidence, for the four species under consideration.

Methods	Human	Mouse	Fly	Yeast
Biochemical assay (MI:0401)	364	109	25	2857
Protein complementation assay (MI:0090)	304	101	20	636
Imaging techniques (MI:0428)	67	26	7	16
Biophysical assay (MI:0013)	48	5	3	82
Others	39	--	--	2252
