# Supplementary material for: Protein-protein interaction as a predictor of subcellular location
Source: BMC Syst Biol. 2009 Feb 25;3:28. doi: 10.1186/1752-0509-3-28 (PMC2663780; doi:10.1186/1752-0509-3-28)
Supplement: Additional file 6 — Evaluation of prediction method variants on reference and supported subsets for human, mouse, fly and yeast. The permissive accuracy (PA) and strict accuracy (SA) were calculated for all variants (DISCRETE, MERGED, COMMON and MAJORITY) for all interactions, and for interactions involving an integral membrane protein (M). [file 1752-0509-3-28-S6.pdf]

**Additional file 6 - Evaluation of prediction method variants on reference and supported subsets for human, mouse, fly and yeast**

The permissive accuracy (PA) and strict accuracy (SA) were calculated for all variants (DISCRETE, MERGED, COMMON and MAJORITY) for all interactions, and for interactions involving an integral membrane protein (M).

| **Human** | **DISCRETE** | | | | **MERGED** | | | | **COMMON** | | | | **MAJORITY** | | | |
|---|---|---|---|---|---|---|---|---|---|---|---|---|---|---|---|---|
| **PPI sets** | **PA** | **SA** | **PA (M)** | **SA (M)** | **PA** | **SA** | **PA (M)** | **SA (M)** | **PA** | **SA** | **PA (M)** | **SA (M)** | **PA** | **SA** | **PA (M)** | **SA (M)** |
| Reference | 0.64 | 0.35 | 0.50 | 0.27 | 0.72 | 0.26 | 0.62 | 0.23 | 0.65 | 0.40 | 0.53 | 0.32 | 0.68 | 0.34 | 0.58 | 0.27 |
| BPscore | 0.84 | 0.56 | 0.76 | 0.46 | 0.86 | 0.55 | 0.79 | 0.42 | 0.85 | 0.57 | 0.77 | 0.47 | 0.86 | 0.55 | 0.79 | 0.43 |
| Interologs | 0.85 | 0.58 | 0.70 | 0.46 | 0.84 | 0.54 | 0.69 | 0.42 | 0.83 | 0.57 | 0.67 | 0.45 | 0.84 | 0.56 | 0.69 | 0.45 |
| DDI | 0.72 | 0.46 | 0.65 | 0.43 | 0.75 | 0.43 | 0.70 | 0.42 | 0.72 | 0.48 | 0.66 | 0.49 | 0.74 | 0.45 | 0.69 | 0.44 |
| PMID | 0.76 | 0.46 | 0.60 | 0.28 | 0.76 | 0.42 | 0.60 | 0.27 | 0.74 | 0.45 | 0.56 | 0.30 | 0.76 | 0.43 | 0.59 | 0.28 |
| Method | 0.74 | 0.38 | 0.61 | 0.29 | 0.76 | 0.37 | 0.63 | 0.27 | 0.73 | 0.41 | 0.58 | 0.32 | 0.75 | 0.38 | 0.62 | 0.29 |
| DB | 0.73 | 0.37 | 0.53 | 0.25 | 0.74 | 0.35 | 0.61 | 0.26 | 0.71 | 0.40 | 0.57 | 0.30 | 0.73 | 0.37 | 0.59 | 0.28 |
| ALL | 0.74 | 0.42 | 0.61 | 0.35 | 0.78 | 0.38 | 0.69 | 0.33 | 0.74 | 0.45 | 0.64 | 0.40 | 0.76 | 0.41 | 0.67 | 0.36 |
| BIO | 0.77 | 0.49 | 0.67 | 0.43 | 0.79 | 0.45 | 0.71 | 0.38 | 0.77 | 0.50 | 0.67 | 0.45 | 0.78 | 0.47 | 0.70 | 0.41 |
| EVI | 0.74 | 0.37 | 0.55 | 0.25 | 0.75 | 0.34 | 0.62 | 0.25 | 0.71 | 0.41 | 0.57 | 0.30 | 0.74 | 0.37 | 0.61 | 0.27 |

| **Mouse** | **DISCRETE** | | | | **MERGED** | | | | **COMMON** | | | | **MAJORITY** | | | |
|---|---|---|---|---|---|---|---|---|---|---|---|---|---|---|---|---|
| **PPI sets** | **PA** | **SA** | **PA (M)** | **SA (M)** | **PA** | **SA** | **PA (M)** | **SA (M)** | **PA** | **SA** | **PA (M)** | **SA (M)** | **PA** | **SA** | **PA (M)** | **SA (M)** |
| Reference | 0.69 | 0.34 | 0.57 | 0.23 | 0.74 | 0.31 | 0.64 | 0.20 | 0.72 | 0.36 | 0.63 | 0.25 | 0.73 | 0.33 | 0.63 | 0.22 |
| BPscore | 0.86 | 0.56 | 0.73 | 0.25 | 0.86 | 0.53 | 0.72 | 0.19 | 0.86 | 0.54 | 0.71 | 0.22 | 0.86 | 0.54 | 0.72 | 0.22 |
| Interologs | 0.77 | 0.42 | 0.60 | 0.20 | 0.79 | 0.41 | 0.60 | 0.16 | 0.78 | 0.43 | 0.59 | 0.20 | 0.79 | 0.43 | 0.60 | 0.20 |
| DDI | 0.82 | 0.40 | 0.75 | 0.33 | 0.83 | 0.36 | 0.77 | 0.26 | 0.82 | 0.38 | 0.77 | 0.29 | 0.82 | 0.37 | 0.77 | 0.27 |
| PMID | 0.82 | 0.43 | 0.81 | 0.34 | 0.83 | 0.40 | 0.81 | 0.24 | 0.82 | 0.42 | 0.81 | 0.31 | 0.82 | 0.41 | 0.81 | 0.31 |
| Method | 0.68 | 0.37 | 0.52 | 0.20 | 0.71 | 0.34 | 0.62 | 0.19 | 0.71 | 0.39 | 0.62 | 0.27 | 0.70 | 0.36 | 0.59 | 0.23 |
| DB | 0.78 | 0.43 | 0.79 | 0.46 | 0.82 | 0.40 | 0.82 | 0.39 | 0.82 | 0.44 | 0.81 | 0.46 | 0.82 | 0.41 | 0.82 | 0.42 |
| ALL | 0.77 | 0.41 | 0.68 | 0.30 | 0.80 | 0.37 | 0.73 | 0.23 | 0.79 | 0.41 | 0.72 | 0.30 | 0.79 | 0.38 | 0.72 | 0.26 |
| BIO | 0.82 | 0.45 | 0.72 | 0.30 | 0.83 | 0.42 | 0.73 | 0.23 | 0.82 | 0.45 | 0.72 | 0.27 | 0.83 | 0.43 | 0.73 | 0.25 |
| EVI | 0.75 | 0.38 | 0.71 | 0.33 | 0.78 | 0.35 | 0.78 | 0.29 | 0.77 | 0.40 | 0.79 | 0.37 | 0.77 | 0.37 | 0.77 | 0.33 |

| Fly | DISCRETE | | | | MERGED | | | | COMMON | | | | MAJORITY | | | |
|---|---|---|---|---|---|---|---|---|---|---|---|---|---|---|---|---|
| PPI sets | PA | SA | PA (M) | SA (M) | PA | SA | PA (M) | SA (M) | PA | SA | PA (M) | SA (M) | PA | SA | PA (M) | SA (M) |
| Reference | 0.57 | 0.32 | 0.50 | 0.22 | 0.65 | 0.28 | 0.61 | 0.20 | 0.61 | 0.38 | 0.59 | 0.30 | 0.62 | 0.32 | 0.58 | 0.23 |
| BPscore | 0.97 | 0.65 | 0.78 | 0.21 | 0.98 | 0.63 | 0.80 | 0.20 | 0.98 | 0.68 | 0.79 | 0.30 | 0.98 | 0.64 | 0.80 | 0.20 |
| Interologs | 0.91 | 0.64 | - | - | 0.91 | 0.62 | - | - | 0.91 | 0.65 | - | - | 0.91 | 0.62 | - | - |
| DDI | 0.94 | 0.65 | 0.89 | 0.39 | 0.95 | 0.62 | 0.93 | 0.40 | 0.94 | 0.66 | 0.87 | 0.40 | 0.95 | 0.65 | 0.93 | 0.40 |
| PMID | 0.91 | 0.57 | 0.76 | 0.12 | 0.93 | 0.51 | 0.85 | 0.06 | 0.92 | 0.60 | 0.75 | 0.17 | 0.93 | 0.56 | 0.85 | 0.06 |
| Method | 0.89 | 0.48 | 0.34 | - | 0.88 | 0.41 | 0.34 | - | 0.86 | 0.48 | 0.34 | - | 0.88 | 0.45 | 0.34 | - |
| DB | 0.47 | 0.27 | 0.29 | 0.21 | 0.56 | 0.27 | 0.39 | 0.28 | 0.51 | 0.33 | 0.38 | 0.35 | 0.53 | 0.29 | 0.35 | 0.29 |
| ALL | 0.56 | 0.32 | 0.41 | 0.18 | 0.65 | 0.30 | 0.53 | 0.21 | 0.61 | 0.38 | 0.52 | 0.27 | 0.62 | 0.33 | 0.49 | 0.21 |
| BIO | 0.93 | 0.60 | 0.75 | 0.20 | 0.95 | 0.57 | 0.85 | 0.21 | 0.94 | 0.63 | 0.84 | 0.28 | 0.94 | 0.60 | 0.82 | 0.21 |
| EVI | 0.51 | 0.28 | 0.33 | 0.17 | 0.60 | 0.27 | 0.43 | 0.20 | 0.55 | 0.34 | 0.42 | 0.25 | 0.57 | 0.30 | 0.40 | 0.20 |

| Yeast | DISCRETE | | | | MERGED | | | | COMMON | | | | MAJORITY | | | |
|---|---|---|---|---|---|---|---|---|---|---|---|---|---|---|---|---|
| PPI sets | PA | SA | PA (M) | SA (M) | PA | SA | PA (M) | SA (M) | PA | SA | PA (M) | SA (M) | PA | SA | PA (M) | SA (M) |
| Reference | 0.60 | 0.34 | 0.45 | 0.23 | 0.77 | 0.15 | 0.58 | 0.10 | 0.59 | 0.36 | 0.36 | 0.23 | 0.67 | 0.35 | 0.46 | 0.23 |
| BPscore | 0.94 | 0.69 | 0.89 | 0.58 | 0.95 | 0.53 | 0.92 | 0.50 | 0.94 | 0.69 | 0.91 | 0.65 | 0.94 | 0.65 | 0.91 | 0.59 |
| Interologs | 0.91 | 0.61 | 1.00 | 0.71 | 0.93 | 0.60 | 1.00 | 0.71 | 0.93 | 0.63 | 1.00 | 0.71 | 0.93 | 0.61 | 1.00 | 0.71 |
| DDI | 0.83 | 0.56 | 0.84 | 0.56 | 0.89 | 0.51 | 0.91 | 0.53 | 0.88 | 0.60 | 0.87 | 0.60 | 0.88 | 0.55 | 0.90 | 0.57 |
| PMID | 0.86 | 0.58 | 0.84 | 0.55 | 0.90 | 0.50 | 0.86 | 0.46 | 0.89 | 0.62 | 0.86 | 0.55 | 0.89 | 0.57 | 0.86 | 0.52 |
| Method | 0.81 | 0.53 | 0.63 | 0.35 | 0.87 | 0.36 | 0.68 | 0.23 | 0.84 | 0.55 | 0.65 | 0.34 | 0.84 | 0.49 | 0.64 | 0.31 |
| DB | 0.62 | 0.36 | 0.43 | 0.22 | 0.74 | 0.19 | 0.48 | 0.13 | 0.60 | 0.36 | 0.36 | 0.20 | 0.67 | 0.34 | 0.42 | 0.18 |
| ALL | 0.66 | 0.40 | 0.51 | 0.29 | 0.77 | 0.21 | 0.56 | 0.17 | 0.64 | 0.40 | 0.44 | 0.28 | 0.71 | 0.39 | 0.50 | 0.25 |
| BIO | 0.92 | 0.66 | 0.88 | 0.57 | 0.94 | 0.50 | 0.92 | 0.50 | 0.93 | 0.67 | 0.90 | 0.64 | 0.93 | 0.62 | 0.90 | 0.58 |
| EVI | 0.63 | 0.37 | 0.44 | 0.23 | 0.75 | 0.20 | 0.49 | 0.14 | 0.61 | 0.37 | 0.37 | 0.21 | 0.67 | 0.35 | 0.43 | 0.19 |

ALL: Union of all subsets (BPscore ∪ Interologs ∪ DDI ∪ PMID ∪ Method ∪ DB)
BIO: Union of sequence-feature-based subsets (BPscore ∪ Interologs ∪ DDI)
EVI: Union of PPI detection processes based subsets (PMID ∪ Method ∪ DB)
M: Interactions involving an integral membrane protein
