# Supplementary material for: Protein-protein interaction as a predictor of subcellular location
Source: BMC Syst Biol. 2009 Feb 25;3:28. doi: 10.1186/1752-0509-3-28 (PMC2663780; doi:10.1186/1752-0509-3-28)
Supplement: Additional file 7 — Interactions between Type I integral membrane proteins and soluble intracellular proteins in human. The PPIs in adjacent compartments (Type I integral membrane proteins and soluble intracellular proteins) are listed for human; only domains present as interacting pairs in the iPfam subset are included. [file 1752-0509-3-28-S7.pdf]

Additional file 7 - Interactions between Type I integral membrane proteins and soluble intracellular proteins in human

In this table, protein_1 represents the integral membrane protein partner and protein_2 the soluble protein partner. TM indicates the trans-membrane region (residue numbers) predicted for protein_1. Pfam_1 and Pfam_2 represent the domains annotated in UniProt for protein_1 and protein_2 respectively; only domains present as interacting pairs in the iPfam subset are included. Pfam residues_1 and _2 indicate the location of these domains in the two protein sequences. Location_1 and _2 represent the SCL of the two proteins as annotated in UniProt. Interacting proteins are considered to represent an adjacent PPI if the topology of the integral membrane protein places the interacting domain in the same compartment as the soluble protein. The "?" indicates that the topology of the relationship cannot be definitively resolved based on the annotated SCL. Cyt, cytoplasm; End, endosome; ExR, extracellular region; Gol, Golgi apparatus; Nuc, nucleus; PM, plasma membrane.

Protein_1	Protein_2	TM_1	Pfam_1	Pfam residues_1	Pfam_2	Pfam residues_2	Location_1	Location_2	Adjacency
P06213	O14544	957-979	PF07714	1023-1290	PF00017	384-461	PM	Cyt	adjacent PPI
Q16288	O60880	434-453	PF07714	538-824	PF00017	7-87	PM	Cyt	adjacent PPI
P04629	O60880	419-439	PF07714	510-781	PF00017	7-87	PM	Cyt	adjacent PPI
Q16620	O60880	433-454	PF07714	538-807	PF00017	7-87	PM	Cyt	adjacent PPI
P14778	P01583	341-359	PF00047	43-98	PF00340	135-266	PM	ExR	adjacent PPI
P14778	P01584	341-359	PF00047	43-98	PF00340	126-264	PM	ExR	adjacent PPI
P04629	P62993	419-439	PF07714	510-781	PF00017, PF00018	60-135, 159-213	PM	Cyt	adjacent PPI
P06213	P29353	957-979	PF07714	1023-1290	PF00017	488-559	PM	PM	?
P06213	Q13322	957-979	PF07714	1023-1290	PF08947, PF00017	423-472, 493-574	PM	Cyt, PM	adjacent PPI
P06213	P41240	957-979	PF07714	1023-1290	PF00017, PF00018, PF07714	82-156, 25-173, 195-440	PM	Cyt, PM	adjacent PPI
P30530	P06239	445-465	PF07714	529-796	PF00018, PF00017, PF07714	64-119, 127-209, 245-494	PM	Cyt, PM	adjacent PPI
Q02763	P06239	746-770	PF07714	824-1092	PF00018, PF00017, PF07714	64-119, 127-209, 245-494	PM	Cyt, PM	adjacent PPI
P07766	P06239	128-150	PF02189	185-205	PF00017	127-209	PM	Cyt, PM	adjacent PPI
P07333	P62993	515-537	PF07714	582-910	PF00017, PF00018	60-135, 159-213	PM	Cyt	adjacent PPI
P07766	P43405		PF02189	185-205	PF00017	168-244	PM	PM	?
P07766	P43403	128-150	PF02189	185-205	PF00017	163-239	PM	Cyt, PM	adjacent PPI
P07766	P29353	128-150	PF02189	185-205	PF00017	488-559	PM	PM	?

P08581	P62993	934-953	PF07714	1078-1337	PF00017, PF00018	60-135, 159-213	PM	Cyt	adjacent PPI
P08581	P40763	934-953	PF07714	1078-1337	PF00017	584-674	PM	Nuc, Cyt	adjacent PPI
P08581	P29353	934-953	PF07714	1078-1337	PF00017	488-559	PM	PM	?
P22455	P09038	373-389	PF00047, PF07679	65-103, 153-241	PF00167	28-149	PM	ExR	adjacent PPI
P11362	P09038	376-396	PF00047, PF07679	48-103, 255-358	PF00167	28-149	PM	ExR	adjacent PPI
P22607	P09038	374-395	PF00047, PF07679	54-111, 157-245	PF00167	28-149	PM	ExR	adjacent PPI
P33151	P14923	597-620	PF01049	621-777	PF02985, PF00514	137-173, 613-653	PM	Cyt	adjacent PPI
P16234	P46108	525-548	PF07714	593-950	PF00017, PF00018	13-104, 135-190	PM	Nuc, Cyt	adjacent PPI
P17948	P29353	764-778	PF07714	827-1154	PF00017	488-559	ExR, PM	PM	?
P18433	P62993	143-166	PF00102	558-790	PF00017	60-135	PM	Cyt	adjacent PPI
P20645	Q9NZ52	186-210	PF02157	1-277	PF00790	3-142	End, PM	Gol	?
P20645	Q9UJY5	186-210	PF02157	1-277	PF00790	5-143	End, PM	Gol	?
P21860	P29353	642-665	PF07714	709-965	PF00017	488-559	PM	PM	?
P22607	P62993	374-395	PF07714	472-748	PF00017, PF00018	60-135, 159-213	PM	Cyt	adjacent PPI
P29317	P62993	536-556	PF07714	613-871	PF00017, PF00018	60-135, 159-213	PM	Cyt	adjacent PPI
P29317	P29353	536-556	PF07714	613-871	PF00017	488-559	PM	PM	?
P29320	P46108	542-565	PF07714	621-878	PF00017, PF00018	13-104, 135-190	PM	Nuc, Cyt	adjacent PPI
Q9UM73	P29353	1036-1056	PF07714	1116-1383	PF00017	488-559	PM	PM	?
P35968	P29353	765-786	PF07714	834-1160	PF00017	488-559	PM	PM	?
Q16288	P29353	434-453	PF07714	538-824	PF00017	488-559	PM	PM	?
Q16832	P29353	400-421	PF07714	563-849	PF00017	488-559	PM	PM	?
P30530	P62993	445-465	PF07714	529-796	PF00017, PF00018	60-135, 159-213	PM	Cyt	adjacent PPI
P30530	P41240	445-465	PF07714	529-796	PF00018, PF00017, PF07714	12-68, 82-156, 195-440	PM	Cyt, PM	adjacent PPI
P33151	P35222	597-620	PF01049	621-777	PF02985, PF00514	146-182, 624-664	PM	Nuc, PM	?
P33151	Q99569	597-620	PF01049	621-777	PF00514	814-855	PM	PM	?
P55291	P35222	595-626	PF01049	629-783	PF02985, PF00514	146-182, 624-664	PM	Nuc, PM	?
Q86UP0	P35222	638-661	PF01049	663-814	PF02985, PF00514	146-182, 624-664	PM	Nuc, PM	?
P35916	P62993	776-798	PF07714	845-1169	PF00017, PF00018	60-135, 159-213	PM	Cyt	adjacent PPI

P35968	P62993	765-786	PF07714	834-1160	PF00017, PF00018	60-135, 159-213	PM	Cyt	adjacent PPI
P35968	Q13322	765-786	PF07714	834-1160	PF08947, PF00017	423-472, 493-574	PM	Cyt, PM	adjacent PPI
P54753	P46108	562-581	PF07714	633-892	PF00017, PF00018	13-104, 135-190	PM	Nuc, Cyt	adjacent PPI
Q14118	P46939	754-775	PF05454	42-895	PF00397, PF09068, PF09069	2814-2843, 2844-2964, 2968-3059	ExR, PM, ExM	PM	?
Q13873	P53667	153-171	PF00069	203-501	PF00069	339-539	PM	Cyt	adjacent PPI
P54762	P62993	545-561	PF07714	619-878	PF00017, PF00018	60-135, 159-213	PM	Cyt	adjacent PPI
P54762	Q13322	545-561	PF07714	619-878	PF08947, PF00017	423-472, 493-574	PM	Cyt, PM	adjacent PPI
Q04912	P62993	958-978	PF07714	1082-1341	PF00017, PF00018	60-135, 159-213	PM	Cyt	adjacent PPI
Q02763	P62993	746-770	PF07714	824-1092	PF00017, PF00018	60-135, 159-213	PM	Cyt	adjacent PPI
Q12866	P62993	502-524	PF07714	587-854	PF00017, PF00018	60-135, 159-213	PM	Cyt	adjacent PPI
Q15303	P78352	653-675	PF07714	718-974	PF00018	431-475	PM	Cyt	adjacent PPI
Q15303	Q12959	653-675	PF07714	718-974	PF00018	584-627	PM	PM	?
Q9HB29	Q14116	337-355	PF00047	35-97	PF00340	56-185	PM	ExR	adjacent PPI
Q14118	Q96QZ7	754-775	PF05454	42-895	PF00397	361-390	ExR, PM, ExM	PM	?
Q15303	Q15700	653-675	PF07714	718-974	PF00018	539-583	PM	PM	?
