# Supplementary material for: Protein-protein interaction as a predictor of subcellular location
Source: BMC Syst Biol. 2009 Feb 25;3:28. doi: 10.1186/1752-0509-3-28 (PMC2663780; doi:10.1186/1752-0509-3-28)
Supplement: Additional file 8 — Evaluation of SCL prediction methods using human reference set and subsets. SCL prediction methods, including three variants of our approach (COMMON, MAJORITY, MERGED) and four existing methods were compared using the human reference set (REF) and two different unions of subsets (BIO and EVI). [file 1752-0509-3-28-S8.pdf]

Additional file 8.1 – Evaluation of prediction method variants using human REF set

PPI sets	COMMON	MAJORITY	MERGED	Proteome Analyst	WolfP SORT	CELLO	pTARGET
**Membrane-membrane PPI only (256)**							
Total # of predicted proteins	226	256	256	55	256	256	120
# of correctly predicted proteins in PA	172	195	199	29	137	140	66
# of correctly predicted proteins in SA	133	129	116	12	102	84	51
PA	0.76	0.76	0.78	0.53	0.54	0.55	0.55
SA	0.59	0.50	0.45	0.22	0.40	0.33	0.43
**Soluble-soluble PPI only (1280)**							
Total # of predicted proteins	864	1253	1280	660	1279	1278	819
# of correctly predicted proteins in PA	632	951	1032	534	780	919	468
# of correctly predicted proteins in SA	409	501	341	345	514	565	309
PA	0.73	0.76	0.81	0.81	0.61	0.72	0.57
SA	0.47	0.40	0.27	0.52	0.40	0.44	0.38
**Membrane-soluble PPI only (491)**							
Total # of predicted proteins	298	319	322	85	322	322	150
# of correctly predicted proteins in PA	85	119	133	63	190	203	84
# of correctly predicted proteins in SA	36	32	33	40	143	130	64
PA	0.29	0.37	0.41	0.74	0.59	0.63	0.56
SA	0.12	0.10	0.10	0.47	0.44	0.40	0.43
**Total (1858)**							
Total # of predicted proteins	1388	1828	1858	800	1857	1856	1089
# of correctly predicted proteins in PA	889	1265	1364	626	1107	1262	618
# of correctly predicted proteins in SA	578	662	490	397	759	779	424
PA	0.64	0.69	0.73	0.78	0.60	0.68	0.57
SA	0.42	0.36	0.26	0.50	0.41	0.42	0.39

* Numbers in the parentheses indicate total number of proteins for prediction for each category

Additional file 8.2 – Evaluation of prediction method variants using human BIO set

PPI sets	COMMON	MAJORITY	MERGED	Proteome Analyst	WolfP SORT	CELLO	pTARGET
**Membrane-membrane PPI only (130)**							
Total # of predicted proteins	130	130	130	34	130	130	65
# of correctly predicted proteins in PA	120	120	120	21	70	76	45
# of correctly predicted proteins in SA	86	81	77	9	49	40	32
PA	0.92	0.92	0.92	0.62	0.54	0.58	0.69
SA	0.66	0.62	0.59	0.26	0.38	0.31	0.49
**Soluble-soluble PPI only (607)**							
Total # of predicted proteins	547	607	607	310	607	607	370
# of correctly predicted proteins in PA	462	523	529	261	377	456	238
# of correctly predicted proteins in SA	301	317	290	154	231	278	150
PA	0.84	0.86	0.87	0.84	0.62	0.75	0.64
SA	0.55	0.52	0.48	0.50	0.38	0.46	0.41
**Membrane-soluble PPI only (144)**							
Total # of predicted proteins	135	144	144	41	144	144	66
# of correctly predicted proteins in PA	46	54	58	32	89	85	43
# of correctly predicted proteins in SA	26	25	23	22	69	59	32
PA	0.34	0.38	0.40	0.78	0.62	0.59	0.65
SA	0.19	0.17	0.16	0.54	0.48	0.41	0.48
**Total (881)**							
Total # of predicted proteins	812	881	881	385	881	881	501
# of correctly predicted proteins in PA	628	697	707	314	536	617	326
# of correctly predicted proteins in SA	413	423	390	185	349	377	214
PA	0.77	0.79	0.80	0.82	0.61	0.70	0.65
SA	0.51	0.48	0.44	0.48	0.40	0.43	0.43

* Numbers in the parentheses indicate total number of proteins for prediction for each category

Additional file 8.3 – Evaluation of prediction method variants using human EVI set

PPI sets	COMMON	MAJORITY	MERGED	Proteome Analyst	WolfP SORT	CELLO	pTARGET
**Membrane-membrane PPI only (73)**							
Total # of predicted proteins	67	73	73	8	73	73	36
# of correctly predicted proteins in PA	52	58	59	4	36	44	20
# of correctly predicted proteins in SA	27	29	25	0	21	21	10
PA	0.78	0.79	0.81	0.50	0.49	0.60	0.56
SA	0.40	0.40	0.34	0.00	0.29	0.29	0.28
**Soluble-soluble PPI only (523)**							
Total # of predicted proteins	457	521	523	265	523	523	316
# of correctly predicted proteins in PA	364	429	437	220	329	393	197
# of correctly predicted proteins in SA	218	227	193	137	207	235	118
PA	0.80	0.82	0.84	0.83	0.63	0.75	0.62
SA	0.48	0.44	0.37	0.52	0.40	0.45	0.37
**Membrane-soluble PPI only (152)**							
Total # of predicted proteins	144	151	152	52	152	152	69
# of correctly predicted proteins in PA	60	67	69	38	90	87	39
# of correctly predicted proteins in SA	30	30	29	24	63	51	24
PA	0.42	0.44	0.45	0.73	0.59	0.57	0.57
SA	0.21	0.20	0.19	0.46	0.41	0.34	0.35
**Total (748)**							
Total # of predicted proteins	668	745	748	325	748	748	421
# of correctly predicted proteins in PA	476	554	565	262	455	524	256
# of correctly predicted proteins in SA	275	286	247	161	291	307	152
PA	0.71	0.74	0.76	0.81	0.61	0.70	0.61
SA	0.41	0.38	0.33	0.50	0.39	0.41	0.36

* Numbers in the parentheses indicate total number of proteins for prediction for each category
