# Supplementary material for: Protein-protein interaction as a predictor of subcellular location
Source: BMC Syst Biol. 2009 Feb 25;3:28. doi: 10.1186/1752-0509-3-28 (PMC2663780; doi:10.1186/1752-0509-3-28)
Supplement: Additional file 9 — Evaluation of prediction method variants using LOCSCL. These 146 PPIs come from the mouse reference set and have additional SCL annotation data available from LOCATE [43]. They are here evaluated against LOCATE SCL annotations. Where both interaction partners have LOCSCL data, the randomization process was applied. [file 1752-0509-3-28-S9.pdf]

Additional file 9 – Evaluation of prediction method variants using LOCSCL

These 146 PPIs come from the mouse reference set and have additional SCL annotation data available from LOCATE [43]. They were previously evaluated against UniProt GO CC terms (Section 8) but are here evaluated against LOCATE SCL annotations. Where both interaction partners have LOCSCL data, the randomization process was applied.

PPI sets	DISCRETE		MERGED		COMMON		MAJORITY	
	PA	SA	PA	SA	PA	SA	PA	SA
Reference	0.79	0.38	0.89	0.42	0.87	0.48	0.85	0.45
ALL	0.83	0.46	0.89	0.49	0.88	0.50	0.83	0.47
BIO	0.89	0.52	0.92	0.57	0.91	0.56	0.92	0.54
EVI	0.59	0.41	0.83	0.53	0.82	0.58	0.78	0.50
